# Synthesis, properties, and applications of novel 9,10-diarylanthracene-based D–A type AIEE fluorescent materials

**DOI:** 10.1039/d6ra07630k

**Published:** 2026-09-29

**Authors:** Rui Tian, Xing Zhou, Shunyang Liu, Renjie Wang, Shouzhi Pu

**Affiliations:** a Jiangxi Provincial Key Laboratory of Organic Functional Molecules, Institute of Organic Chemistry, Jiangxi Science and Technology Normal University Nanchang 330013 PR China bio-wrj@163.com pushouzhi@tsinghua.org.cn; b Jiangxi Key Laboratory of Advanced Ceramic Materials, School of Materials Science and Engineering, Jingdezhen Ceramic University Jingdezhen 333403 China

## Abstract

Two novel aggregation-induced emission enhancement (AIEE) active donor–π–acceptor (D–π–A) molecules, namely TP-ATA and CP-ATA, were rationally designed and synthesized. In these two molecules, triphenylamine and carbazole act as electron-donating skeletons, while terminal thiophene-acrylonitrile groups function as electron-withdrawing moieties. Both compounds exhibit distinct solvatochromic behaviors, verifying their intrinsic intramolecular charge transfer (ICT) characteristics. In addition, TP-ATA and CP-ATA possess reversible mechanofluorochromic (MFC) properties. Upon grinding, TP-ATA displays a more prominent fluorescence color transition from bright green to bright yellow, accompanied by an emission redshift of 35 nm, which is more significant than that of CP-ATA. X-ray diffraction characterization confirms that the disturbed molecular packing after grinding can be completely restored *via* fuming with dichloromethane. This molecular fluorophore modulation strategy provides an effective approach to tuning the photophysical properties of anthracene-based D–π–A molecules. Taking advantage of their good solid-state luminescence performances, both molecules have been successfully applied to the visualization of latent fingerprints on various substrates. Furthermore, cellular fluorescence imaging experiments demonstrate that CP-ATA can efficiently penetrate cell membranes to achieve intracellular fluorescence imaging, indicating its promising application potential in the biological field.

## Abstract 2

Two 9,10-diarylanthracene D–π–A AIEE molecules exhibit mechanofluorochromism for high-contrast latent fingerprint visualization.

## Graphical abstract

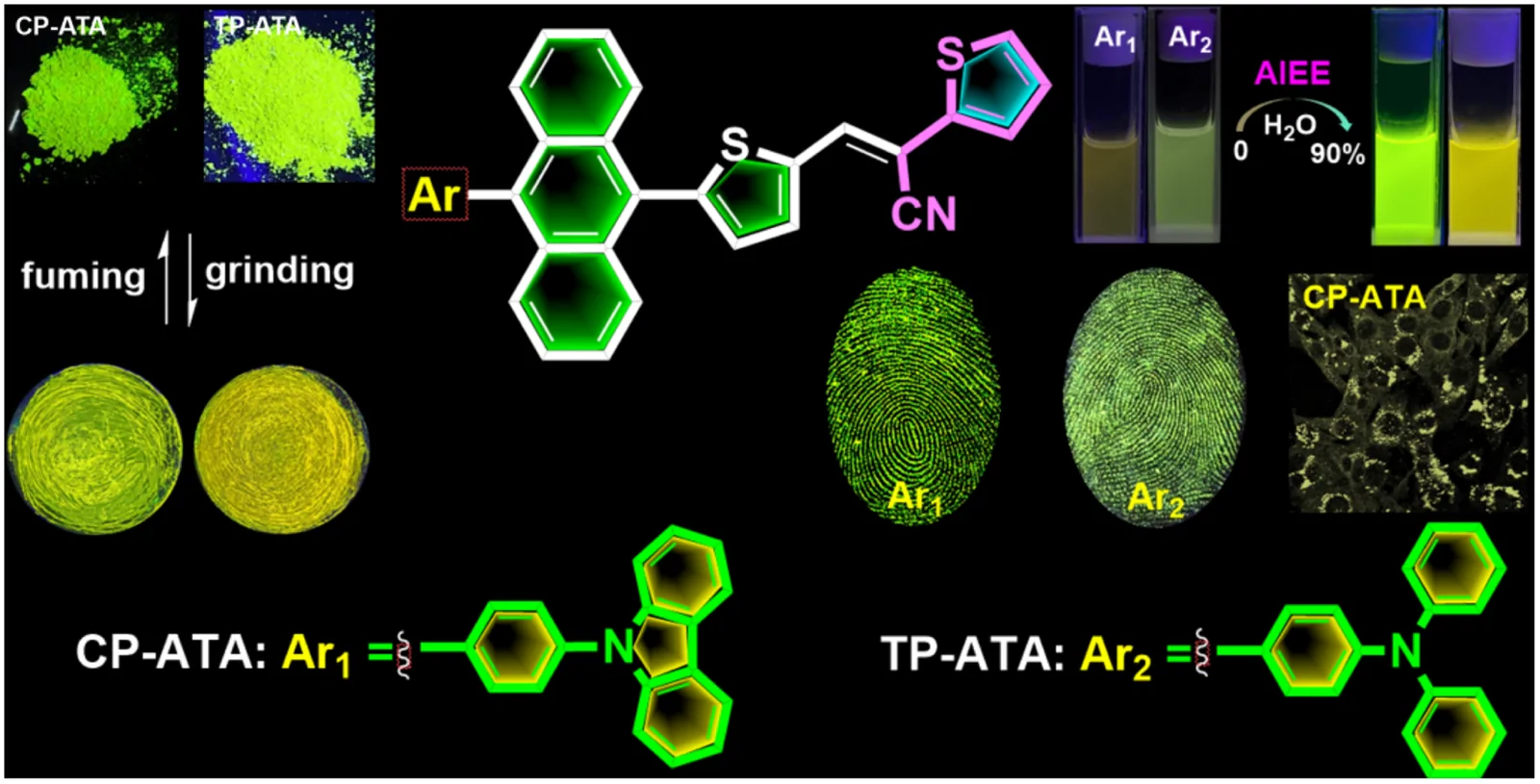

## 1. Introduction

Traditional organic luminescent materials are characterized by tunable molecular structures and favorable biocompatibility, yet they are inevitably affected by aggregation-caused quenching (ACQ). These luminogens exhibit bright emission in dilute solutions; however, their photoluminescence efficiency decreases significantly or is completely quenched in the aggregated and solid states, which greatly restricts their practical application.^1–3^ This intrinsic drawback severely narrows the application scope of conventional organic fluorophores in fields such as solid-state optoelectronic devices and high-concentration biological imaging.^4,5^ To overcome this bottleneck, Tang and co-workers pioneered a breakthrough in 2001, discovering that multiphenyl-substituted silole derivatives exhibited abnormal luminescence behavior with enhanced emission upon aggregation. This unique phenomenon was defined as “aggregation-induced emission” (AIE).^6^ On the basis of extensive experimental and theoretical investigations, the restriction of intramolecular motion (RIM) mechanism was established as the core principle responsible for AIE activity.^7,8^ This fundamental mechanism fundamentally solves the long-standing ACQ problem and provides reliable guidance for the rational design and fabrication of high-performance luminescent materials.^9–11^ Over the past two decades, AIE research has developed from initial phenomenon discovery and mechanistic exploration into a comprehensive research system involving molecular design, photophysical modulation, device fabrication, and multifunctional applications. To date, AIE materials have achieved remarkable advances and been widely exploited in diverse fields, including public security,^12,13^ industrial manufacturing,^14,15^ biomedicine,^16,17^ chemical sensing,^18,19^ as well as agriculture and food industries.^20,21^ Typical AIE-active luminogens, such as tetraphenylethylene (TPE),^22,23^ 9,10-distyrylanthracene (DSA) derivatives,^24^ and cyanostilbene derivatives,^25^ have been extensively developed to construct versatile stimuli-responsive luminescent systems with mechanofluorochromic, photochromic, solvatochromic, and ion-sensing properties.^26–28^

Anthracene is a classic and well-explored fluorophore with excellent photophysical performance among organic luminogens. Its derivatives possess favorable charge transport capability and high photoluminescence quantum yield,^29,30^ rendering them promising candidates for optoelectronic devices such as organic light-emitting transistors (OLETs) and organic light-emitting diodes (OLEDs).^31,32^ Nevertheless, the planar skeleton of anthracene readily induces intense intermolecular π–π stacking in the aggregated and solid states, leading to severe ACQ effect and poor solid-state luminescence. Structural functionalization at the 9,10-positions of anthracene, especially through substitution with styryl or bulky aromatic groups, can introduce steric repulsion against the 1,8-hydrogen atoms and trigger moderate molecular torsion. Such structural distortion effectively suppresses excessive π–π stacking and alleviates ACQ behavior, thereby retaining high luminescence efficiency in the aggregation state. Notably, some modified anthracene derivatives even exhibit unique AIE characteristics.^33,34^ Accordingly, DSA and its derivatives have emerged as a class of promising functional materials for organic optoelectronics.^35,36^ In our previous studies, a series of novel luminophores were constructed by modifying the 9,10-active sites of anthracene with five-membered heterocyclic units. The resulting molecules exhibit prominent AIEE characteristics, large Stokes shift, good biocompatibility, and facile structural tunability, enabling their versatile applications in MFC sensing and intracellular fluorescence imaging.^37,38^ In this context, it is highly desirable to further expand the molecular library of anthracene-based AIEE compounds and explore the structure–photophysics relationship of anthracene derivatives. In particular, rational modification at the 9,10-positions of anthracene with different five-membered heterocycles and benzene rings can effectively regulate molecular rigidity and steric hindrance, tailor molecular spatial conformations and intermolecular stacking modes, and further afford high-performance anthracene-based AIEE luminogens with excellent optical properties.

In this work, two novel D–π–A-structured luminogens, namely TP-ATA and CP-ATA, were rationally designed and synthesized. The molecules adopt triphenylamine or carbazole as electron-donating skeletons, while thiophene-cyano groups serve as electron-withdrawing moieties. The AIEE behavior, solvatochromic effect, and MFC performance of the two compounds were systematically investigated. Benefiting from their good solid-state luminescence, the as-prepared luminogens were successfully applied for latent fingerprint visualization on various substrates. Furthermore, the cell cytotoxicity of them was evaluated *via* the MTT assay, verifying that CP-ATA can efficiently internalize into living cells under physiological conditions and achieve effective cellular fluorescence staining. The molecular structures of CP-ATA and TP-ATA are shown in Fig. 1.

**Fig. 1:**
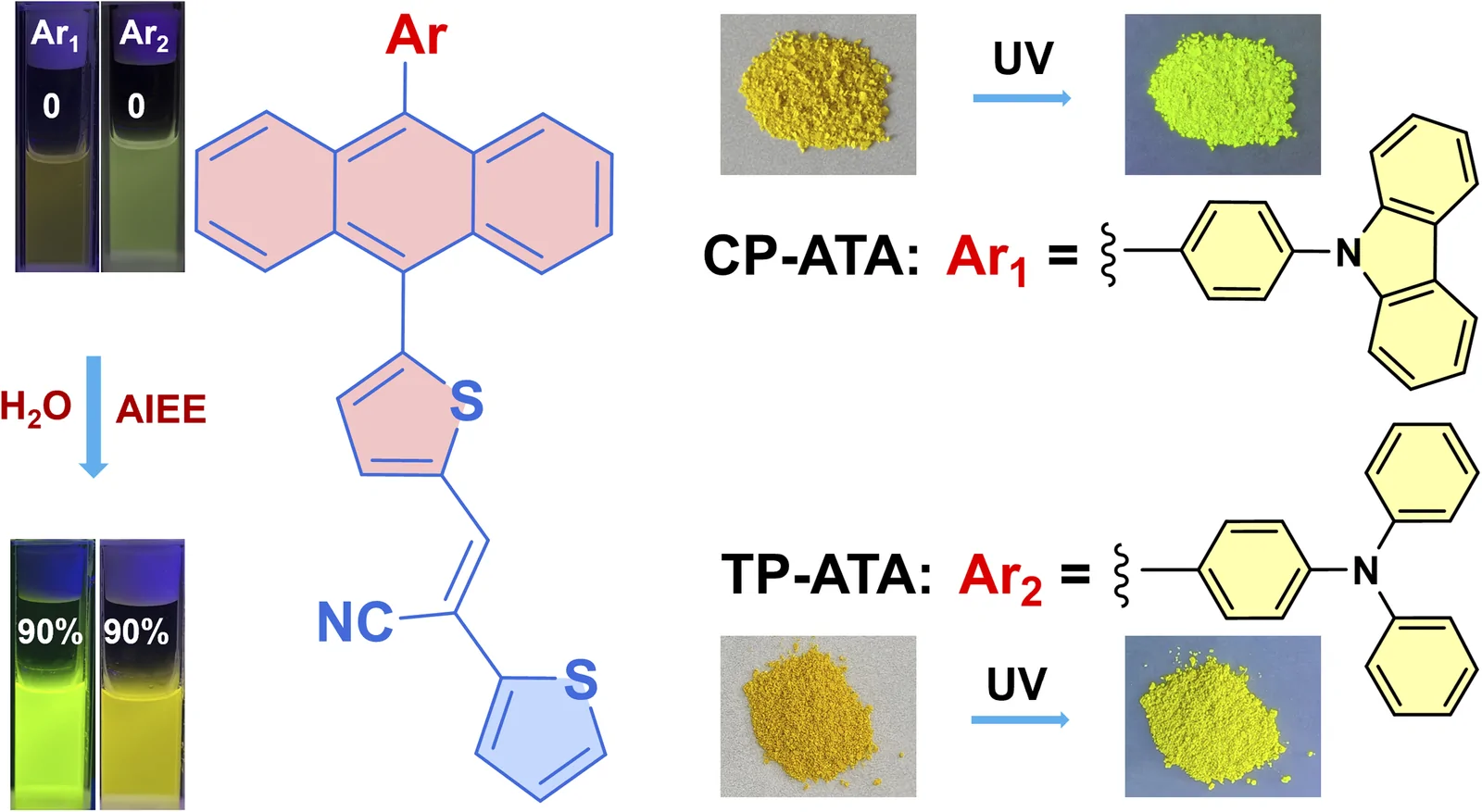
The molecular structures and photographs of CP-ATA and TP-ATA.

## 2. Experimental section

### 2.1. General methods

All chemical reagents and solvents were commercially available and used directly without further purification, including 9,10-dibromoanthracene, 5-formyl-2-thiopheneboronic acid, 2-thiopheneacetonitrile, 4-(9-carbazolyl)phenylboronic acid, 4-triphenylaminophenylboronic acid, anhydrous potassium phosphate, tetrabutylammonium hydroxide, palladium chloride, tetrahydrofuran (THF), dichloromethane (DCM), petroleum ether, ethyl acetate, and ethanol. The ^1^H NMR spectra and ^13^C NMR spectra of the target compounds were recorded on a Bruker AV 500 spectrometer (500 MHz). High-resolution mass spectrometry (HRMS) data were acquired using a Waters Xevo G3 Quadrupole time-of-flight mass spectrometer. UV-vis absorption spectra were measured on an Agilent 8454 spectrophotometer, while steady-state fluorescence spectra were collected with a Hitachi F-4600 fluorescence spectrophotometer. Absolute photoluminescence quantum yields were determined *via* a Hamamatsu C11347-11 Absolute PL Quantum Yield Spectrometer. Melting points were measured using an X-4 microscopic melting point apparatus. Dynamic light scattering (DLS) tests were performed on a Brookhaven Nanobook 90 Plus instrument. Fluorescence lifetime decay curves were obtained *via* an Edinburgh FLS 980 spectrometer. X-ray diffraction (XRD) patterns were recorded on a Malvern Panalytical Empyrean X-ray diffractometer equipped with a Cu K_α_ radiation source and a one-dimensional array detector. The instrument was operated in *θ*/*θ* scanning mode with a goniometer angle reproducibility lower than 0.0001°. The microscopic morphology of the aggregated samples was characterized using a Zeiss Sigma 360 field emission scanning electron microscope (SEM, Germany).

### 2.2. Synthesis

The synthetic routes of CP-ATA and TP-ATA are depicted in Scheme 1. The key intermediate 5-(10-bromoanthracen-9-yl)thiophene-2-carbaldehyde (1) was synthesized according to our group's reported method.^39^ Subsequently, Knoevenagel condensation of intermediate 1 with 2-thiopheneacetonitrile was performed to afford 3-(5-(10-bromoanthracen-9-yl)thiophen-2-yl)-2-(thiophen-2-yl)acrylonitrile (Br-ATA). Finally, utilizing Br-ATA as the crucial precursor, Suzuki cross-coupling reactions with 4-(9-carbazolyl)phenylboronic acid and 4-triphenylaminophenylboronic acid were separately carried out to yield the target compounds CP-ATA and TP-ATA, respectively. The chemical structures of the two synthesized molecules were fully verified by NMR spectroscopy and HRMS, with the corresponding characterization data presented in Fig. S1–S5 of the SI.

**Scheme 1:**
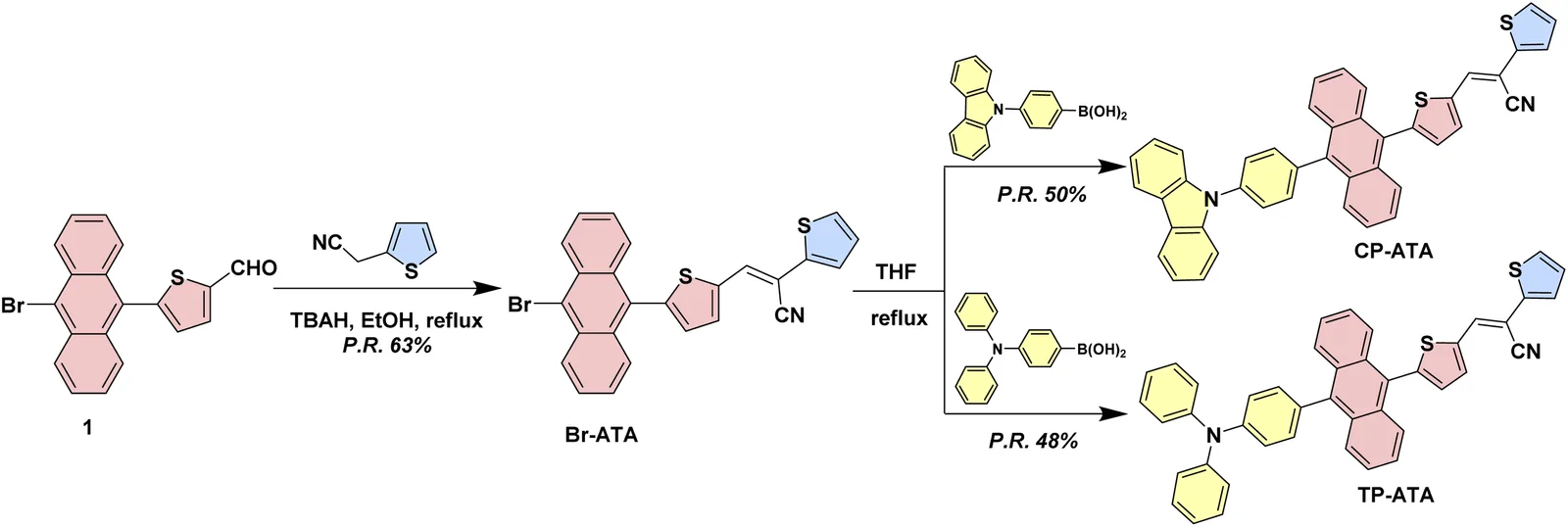
The synthetic routes of Br-ATA, CP-ATA and TP-ATA.

#### 2.2.1. 3-(5-(10-Bromoanthracen-9-yl)thiophen-2-yl)-2-(thiophen-2-yl)acrylonitrile (Br-ATA)

A 50 mL round-bottom flask was fitted with a magnetic stirrer and reflux condenser. 5-(10-Bromoanthracen-9-yl)thiophene-2-carbaldehyde (1.0 g, 0.003 mol), 2-thiopheneacetonitrile (0.3 mL) were dissolved in ethanol, followed by the addition of tetrabutylammonium hydroxide solution as the catalyst. The resulting mixture was heated to 80 °C and vigorously stirred for 6 h to complete the reaction. After cooling naturally to room temperature, a large amount of solid precipitated was formed. The crude product was collected by suction filtration, thoroughly rinsed with ethanol, and dried to obtain the target product Br-ATA as a yellowish-green solid. The final product yield was 63% (0.8 g, 1.7 mmol). The detailed structural characterization data are presented below: mp. 218–219 °C; ^1^H NMR (500 MHz, CDCl_3_): *δ* 8.62 (d, *J* = 9.0 Hz, 2H), 7.88–7.81 (m, 3H), 7.65–7.60 (m, 2H), 7.58 (s, 1H), 7.51–7.46 (m, 2H), 7.37 (s, 1H), 7.33 (d, *J* = 5.0 Hz, 1H), 7.22 (d, *J* = 3.5 Hz, 1H), 7.11–7.08 (m, 1H); ^13^C NMR (126 MHz, CDCl_3_): *δ* 143.6, 139.3, 138.7, 132.3, 132.2, 131.7, 130.8, 130.2, 128.3, 128.0, 127.9, 127.2, 127.2, 126.7, 126.6, 126.2, 125.3, 117.0, 103.4.

#### 2.2.2. 3-(5-(10-(4-(9*H*-Carbazol-9-yl)phenyl)anthracen-9-yl)thiophen-2-yl)-2-(thiophen-2-yl)acrylonitrile (CP-ATA)

A 100 mL three-necked flask was loaded with Br-ATA (0.5 g, 1.1 mmol), 4-(9*H*-carbazol-9-yl)phenylboronic acid (0.4 g, 1.4 mmol), aqueous Na_2_CO_3_ solution (2.0 mol L^−1^, 30.0 mL) and [1,1′-bis(diphenylphosphino)ferrocene]dichloropalladium (0.008 g, 0.01 mmol), followed by the addition of 50.0 mL tetrahydrofuran as the reaction solvent. The sealed reaction system was purged with argon to remove air and moisture, and the mixture was refluxed with continuous stirring for 12 h. Upon cooling to room temperature, the solvent was removed under reduced pressure. The resulting mixture was extracted with dichloromethane and deionized water, and the collected organic phase was dried over anhydrous sodium sulfate, filtered, and concentrated *in vacuo*. The crude product was purified by silica gel column chromatography using petroleum ether/ethyl acetate (10 : 1, v/v) as the eluent. The final product CP-ATA was obtained as a yellow solid with a yield of 50% (0.3 g, 0.5 mmol). The detailed structural characterization data are displayed below: mp. 249–250 °C; ^1^H NMR (500 MHz, CDCl_3_): *δ* 8.22 (d, *J* = 8.0 Hz, 2H), 8.00–7.92 (m, 2H), 7.87–7.80 (m, 5H), 7.69 (m, 4H), 7.62 (s, 1H), 7.54–7.46 (m, 6H), 7.42–7.29 (m, 5H), 7.11–7.09 (m, 1H); ^13^C NMR (126 MHz, CDCl_3_): *δ* 144.5, 140.9, 140.8, 139.8, 139.1, 138.9, 138.2, 138.2, 137.8, 137.6, 137.4, 137.3, 134.9, 132.7, 132.0, 132.5, 132.4, 131.9, 131.3, 131.1, 130.7, 129.9, 129.9, 129.8, 129.8, 129.1, 128.3, 128.0, 127.7, 127.1, 127.0, 126.9, 126.9, 126.5, 126.4, 126.2, 126.1, 126.1, 125.7, 125.7, 123.6, 120.5, 120.2, 120.2; HRMS-ESI (*m*/*z*): [M + H^+^] calcd for C_43_H_27_N_2_S_2_^+^ 635.1610, found 635.1606.

#### 2.2.3. 3-(5-(10-(4-(Diphenylamino)phenyl)anthracen-9-yl)thiophen-2-yl)-2-(thiophen-2-yl)acrylonitrile (TP-ATA)

The synthesis of compound TP-ATA was conducted following a similar procedure as to that used for CP-ATA, resulting in the formation of a yellow solid with a yield of 48% (0.3 g, 0.5 mmol). The structural characterization data are given below: mp. 244–245 °C; ^1^H NMR (500 MHz, CDCl_3_): *δ* 7.86–7.74 (m, 4H), 7.59–7.38 (m, 8H), 7.35 (t, *J* = 7.0 Hz, 4H), 7.32–7.26 (m, 8H), 7.19–7.15 (m, 1H), 7.13–7.07 (m, 3H); ^13^C NMR (126 MHz, CDCl_3_): *δ* 147.6, 147.2, 141.9, 138.8, 136.8, 134.5, 132.0, 131.8, 131.3, 129.8, 129.6, 129.3, 128.6, 127.9, 127.5, 127.2, 127.0, 126.4, 125.7, 125.0, 124.7, 123.1, 122.8, 120.0; HRMS-ESI (*m*/*z*): [M + H^+^] calcd for C_43_H_29_N_2_S_2_^+^ 637.1767, found 637.1763.

## 3. Results and discussion

### 3.1. Solvatochromic properties

The solvent-dependent photophysical properties of CP-ATA and TP-ATA were systematically investigated in eight solvents with gradient polarities, including *n*-hexane, 1,4-dioxane, ethyl acetate (EA), tetrahydrofuran (THF), dimethyl sulfoxide (DMSO), *N*,*N*-dimethylformamide (DMF), acetonitrile (MeCN), and methanol (MeOH), at a consistent concentration of 2.0 × 10^−5^ mol L^−1^. The solvent-variable UV-vis absorption spectra of CP-ATA are presented in Fig. S6A (SI). As the solvent polarity gradually increases from *n*-hexane to DMF, the maximum absorption wavelength (*λ*_abs_) of CP-ATA slightly redshifts from 393 nm to 396 nm. Nevertheless, a subtle blueshift of the absorption peak occurs upon further increasing the solvent polarity to a higher level. In comparison, TP-ATA shows negligible variations in absorption spectra across all tested solvents, indicating that solvent polarity has a limited influence on its ground-state electronic structure (Fig. S6B, SI). In sharp contrast to the weak solvent dependence of absorption, both compounds exhibit prominent solvatochromic fluorescence responses (Fig. 2). For CP-ATA, the maximum emission wavelength (*λ*_em_) continuously redshifts from 550 nm in nonpolar *n*-hexane to 589 nm in moderately polar DMSO. Ultimately, a distinct blueshift to 576 nm is observed in strongly protic MeOH (Fig. 2A). Similarly, TP-ATA displays a more dramatic solvatochromic trend: its emission redshifts significantly from 505 nm (*n*-hexane) to 586 nm (DMSO), and subsequently undergoes a remarkable blueshift to 550 nm in MeOH (Fig. 2B). The overall spectral evolution reveals typical intramolecular charge transfer (ICT) characteristics for both D–π–A luminogens. In common aprotic solvents with low-to-moderate polarity, increased solvent polarity effectively stabilizes the charge-separated ICT excited state, reduces the excited-state energy gap, and thus induces a pronounced emission redshift, which is a classic signature of ICT behavior. However, this conventional solvatochromic rule is reversed in strongly protic methanol, where the redshift tendency is greatly suppressed and an obvious fluorescence blueshift emerges. This anomalous blueshift in protic solvents can be reasonably explained by the inhibition of twisted intramolecular charge transfer (TICT). In polar protic environments, strong intermolecular interactions between CP/TP-ATA and methanol might rigidify the molecular conformation, restrict intramolecular torsional motions, and destroy the twisted molecular geometry required for TICT state formation. Consequently, the generation of the long-wavelength TICT state is effectively suppressed. The excited-state emission is therefore dominated by the short-wavelength localized excited (LE) state rather than the ICT/TICT state. The transition from low-energy TICT emission to high-energy LE emission increases the S_0_–S_1_ energy gap, ultimately resulting in the notable fluorescence blueshift observed in protic solvents.^40^

**Fig. 2:**
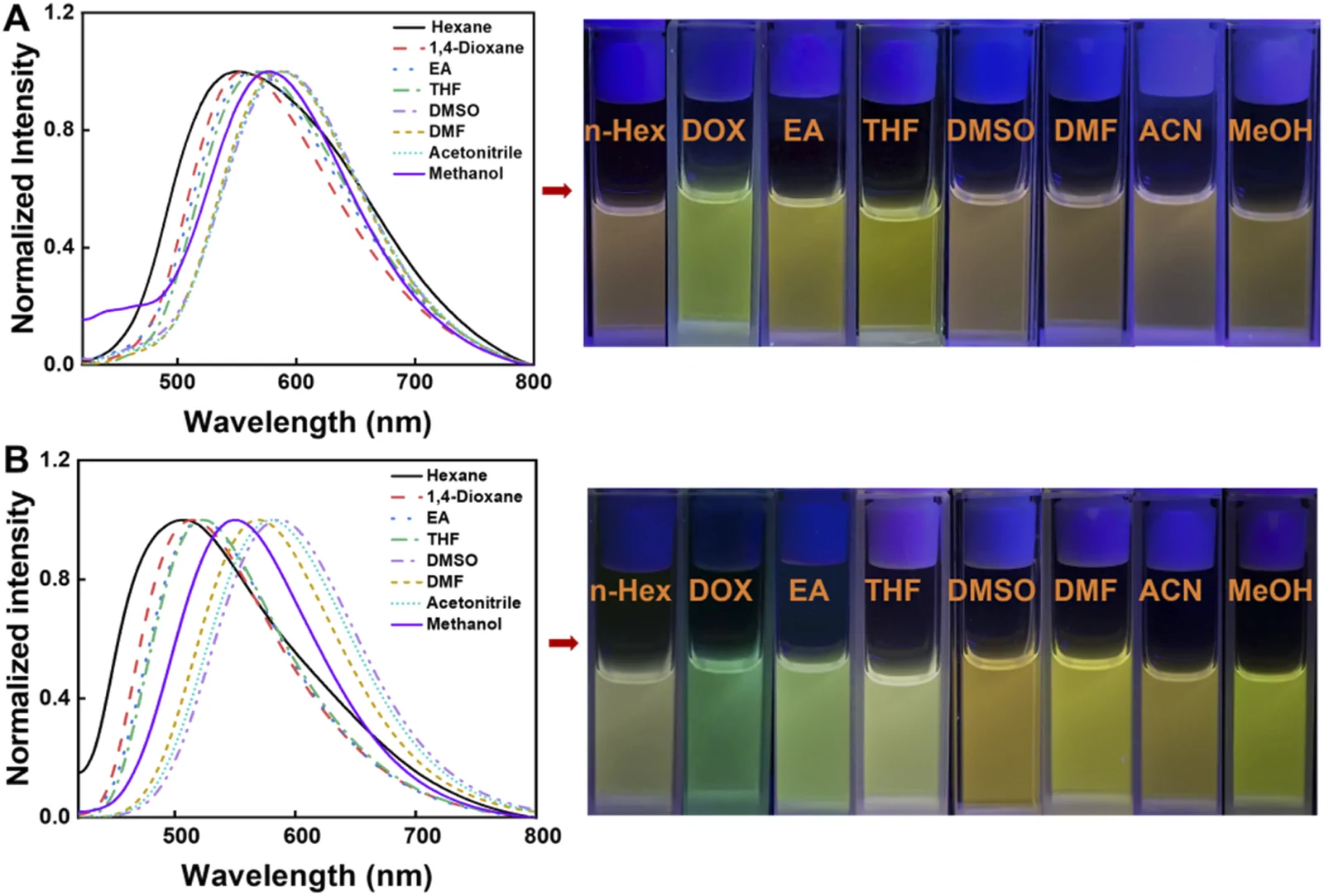
The emission spectra of CP-ATA (A) and TP-ATA (B) in different solvents (*C* = 2.0 × 10^−5^ mol L^−1^) and corresponding photographs under 365 nm UV irradiation.

### 3.2. Photophysical properties

The UV-vis absorption and steady-state fluorescence spectra of CP-ATA and TP-ATA were measured in THF at a concentration of 2.0 × 10^−5^ mol L^−1^, and the corresponding spectra are presented in Fig. 3A and B, respectively. The high-energy absorption bands below 300 nm originate from the π → π* electronic transition of the anthracene conjugated skeleton, while the characteristic absorption peaks centered at approximately 400 nm correspond to ICT transitions from the electron-donating moieties (triphenylamine or carbazole) to the electron-withdrawing cyano group.^41^CP-ATA exhibits a maximum absorption wavelength at 393 nm, which is slightly blueshifted relative to that of TP-ATA (*λ*_abs_ = 397 nm). This difference is ascribed to the relatively weaker electron-donating capability of the carbazole unit compared with triphenylamine. The weakened donor strength reduces the degree of ground-state charge separation, suppresses the ICT effect, and consequently induces a hypsochromic shift in the absorption band. Interestingly, an opposite trend is observed in the fluorescence emission spectra. CP-ATA shows a significantly redshifted emission peak at 567 nm in THF solution, compared with TP-ATA (*λ*_em_ = 523 nm). This distinct emission behavior is closely related to the structural rigidity and molecular planarity of different donor units. As a rigid fused-ring donor, carbazole endows the CP-ATA molecule with higher conjugated planarity. Upon photoexcitation, the rigid molecular skeleton effectively restricts intramolecular rotational and vibrational motions, suppresses non-radiative energy dissipation, and enhances the ICT intensity. In contrast, triphenylamine possesses a typical non-planar propeller-like conformation. The flexible structure allows vigorous intramolecular torsion in the excited state, which consumes partial excitation energy through non-radiative relaxation pathways and ultimately weakens the excited-state ICT behavior, resulting in a shorter emission wavelength for TP-ATA in solution. In the solid state, CP-ATA exhibits an absorption maximum at 384 nm and an emission maximum at 558 nm, while TP-ATA displays absorption and emission peaks at 394 nm and 575 nm, respectively. Both the absorption and emission wavelengths of solid-state TP-ATA are redshifted relative to those of CP-ATA. This phenomenon benefits from the stronger electron-donating ability of triphenylamine, which enhances the intramolecular D–π–A electronic interaction, reduces the excited-state energy gap, and thus induces a bathochromic shift in both absorption and emission. Notably, distinct solid-state spectral variations are observed for the two compounds compared with their solution-state spectra. Specifically, CP-ATA exhibits a solid-state emission blueshift, whereas TP-ATA shows a solid-state emission redshift (Fig. 3C and D), which is mainly governed by their different molecular packing modes in the solid. Fluorescence lifetime decay curves were further recorded to explore the excited-state dynamics (Fig. 3E and F). In THF solution, the fluorescence lifetimes of CP-ATA and TP-ATA are 0.42 ns and 2.62 ns, respectively. In the solid state, their lifetimes are prolonged to 0.67 ns and 0.97 ns. Triphenylamine with a non-planar spatial structure can loosen molecular stacking and inhibit intermolecular π–π interactions, thereby suppressing non-radiative decay and prolonging the fluorescence lifetime.^42^ In comparison, the planar carbazole skeleton might form tighter molecular packing, leading to faster excited-state decay and a shorter lifetime. These results demonstrate that the electronic properties, molecular conformation, and solid-stacking behavior of D–π–A luminogens can be efficiently regulated by modifying electron-donating groups. This structural modulation strategy enables tuning of photophysical performance, providing a reliable guideline for the rational design and optimization of high-performance anthracene-based luminescent materials. The detailed photophysical parameters of CP-ATA and TP-ATA in solution and solid states are summarized in Table 1.

**Fig. 3:**
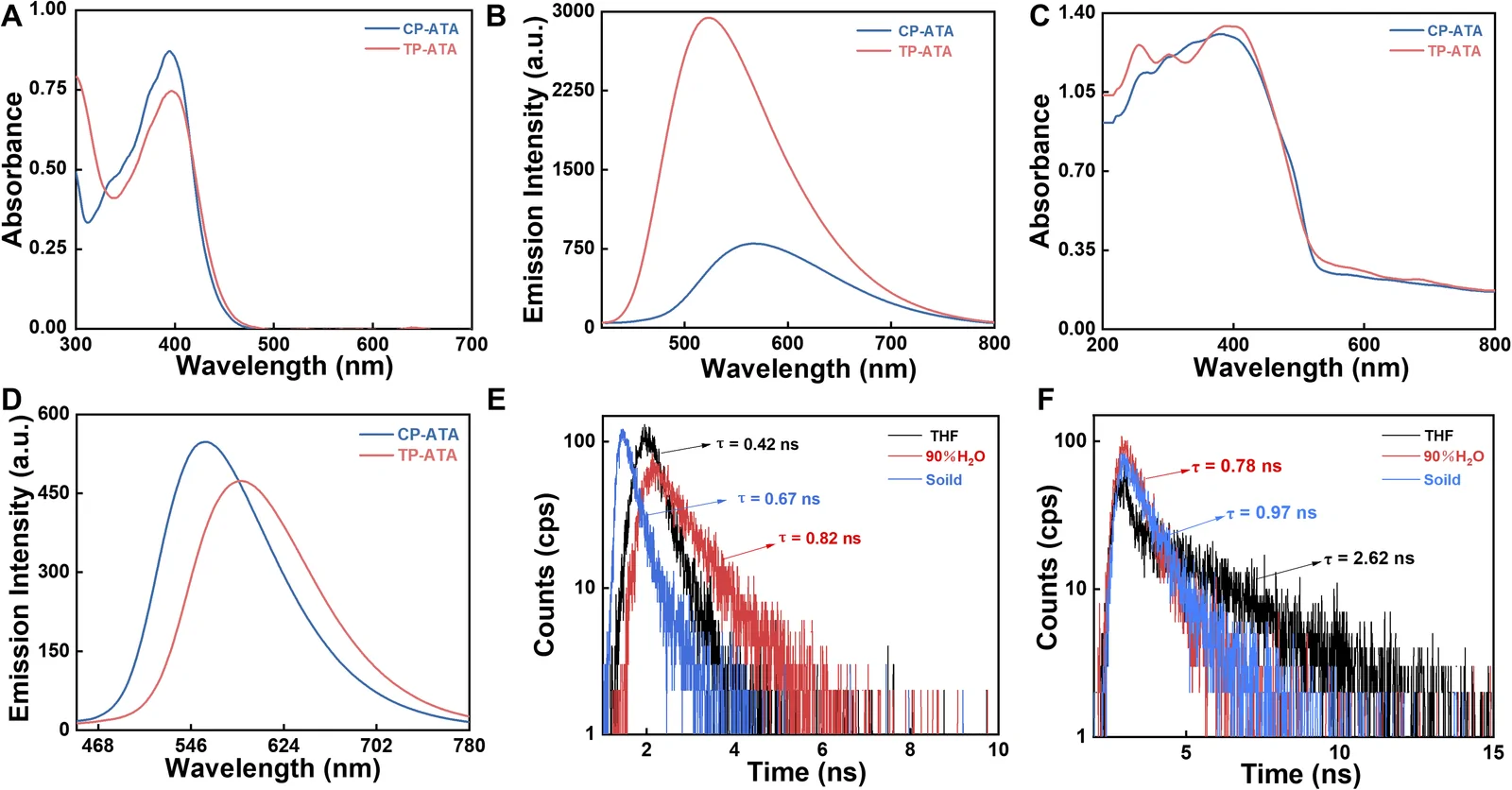
UV-vis absorption and photoluminescence spectra of CP-ATA and TP-ATA in various states: (A and B) in THF solution (*C* = 2.0 × 10^−5^ mol L^−1^), (C and D) in solid state; (E and F) fluorescence lifetimes of CP-ATA and TP-ATA in THF solution, aggregated state, and solid state.

**Table 1:** Photophysical characteristics of CP-ATA and TP-ATA in THF (*C* = 2.0 × 10^−5^ mol L^−1^) and solid states

Comps	THF				Solid			Aggregation states			
	*λ*_max_ a/nm (*ε*/mol^−1^ L cm^−1^)	*λ*_em,max_ b (nm)	*Φ*_f_ c	*τ* d (ns)	*λ*_max_ a (nm)	*λ*_em_ b (nm)	*τ* d (ns)	*λ*_em,max_ b (nm)	H_2_O (%)	*τ* d (ns)	*Φ*_fl_ c
CP-ATA	393 (4.23 × 10^4^)	567	0.013	0.42	384	558	0.67	560	90%	0.82	0.110
TP-ATA	397 (3.74 × 10^4^)	523	0.050	2.62	394	575	0.97	574	90%	0.78	0.086

a Absorption maxima wavelength.

b Emission peak.

c *Φ*_f_ fluorescence quantum yield.

d *τ* fluorescence lifetimes.

### 3.3. Aggregation-induced emission enhancement (AIEE)

To verify the AIEE characteristics of CP-ATA and TP-ATA, their photophysical behaviors were systematically investigated in tetrahydrofuran/water (THF/H_2_O) mixed solvents with gradient water fractions (*f*_w_). For CP-ATA, the absorption profiles remain nearly unchanged when *f*_w_ < 60%. When *f*_w_ increases to 70%, the absorbance reaches its maximum, accompanied by a distinct absorption redshift from 393 nm to 411 nm and an elevated baseline in the long-wavelength region (Fig. S7A, SI). This spectral variation originates from the decreased solvent solubility at high *f*_w_. The deteriorated solvation capacity prevents complete molecular dissolution and triggers the formation of nanoscale aggregates. The resulting suspended aggregates produce an obvious light scattering effect, mainly Mie scattering, leading to baseline elevation.^43^TP-ATA exhibits a similar solvent-dependent absorption evolution upon *f*_w_ increasing (Fig. S7B, SI). The fluorescence emission spectra of CP-ATA in THF/H_2_O mixtures with different *f*_w_ values are presented in Fig. 4A. The emission intensity remains almost constant at *f*_w_ < 60%. When *f*_w_ = 70%, the fluorescence intensity begins to increase significantly and achieves a maximum 10-fold enhancement at *f*_w_ = 90%. Meanwhile, the fluorescence color changes from yellow to bright yellowish-green under 365 nm UV irradiation. In pure THF, free intramolecular rotation and vigorous ICT dominate the excited-state relaxation, which opens abundant non-radiative decay channels and results in weak monomer emission. With the introduction of a large proportion of water, the poor solubility restricts intramolecular motions through molecular aggregation, effectively suppressing non-radiative dissipation and activating aggregation-enhanced emission, which confirms the typical AIEE feature of CP-ATA.^44^ In contrast, TP-ATA displays distinctly different fluorescence evolution trends during aggregation (Fig. 4B). As *f*_w_ increases from 0% to 50%, the emission intensity gradually increases, accompanied by a continuous emission redshift from 523 nm to 550 nm. However, the emission intensity decreases sharply at *f*_w_ = 60–70%. Subsequently, the luminescence intensity rebounds at *f*_w_ = 80% and ultimately reaches the maximum at *f*_w_ = 90% (*λ*_em_, TP-ATA = 574 nm), with the fluorescence color changing from green to bright yellow. This unique aggregation-dependent luminescence behavior of TP-ATA can be reasonably explained by the dynamic evolution of molecular aggregation states. At the initial aggregation stage (*f*_w_: 60–70%), molecules begin to aggregate from the dispersed monomer state but form loose and unstable aggregate structures. The insufficient steric hindrance and disordered molecular arrangement fail to effectively restrict intramolecular motions, and the residual non-radiative decay still dominates excited-state relaxation. Furthermore, the loosely packed aggregates induce intensified intermolecular π–π stacking interactions, which cause severe excited-state quenching and further reduce luminescence efficiency. As the *f*_w_ further increases to 80–90%, the unstable loose aggregates gradually transform into compact and stable nanoaggregates. The tightly confined molecular conformation significantly restricts intramolecular rotations, blocks non-radiative decay pathways, and thereby recovers and enhances the fluorescence emission, exhibiting typical AIEE activation behavior.^45^

**Fig. 4:**
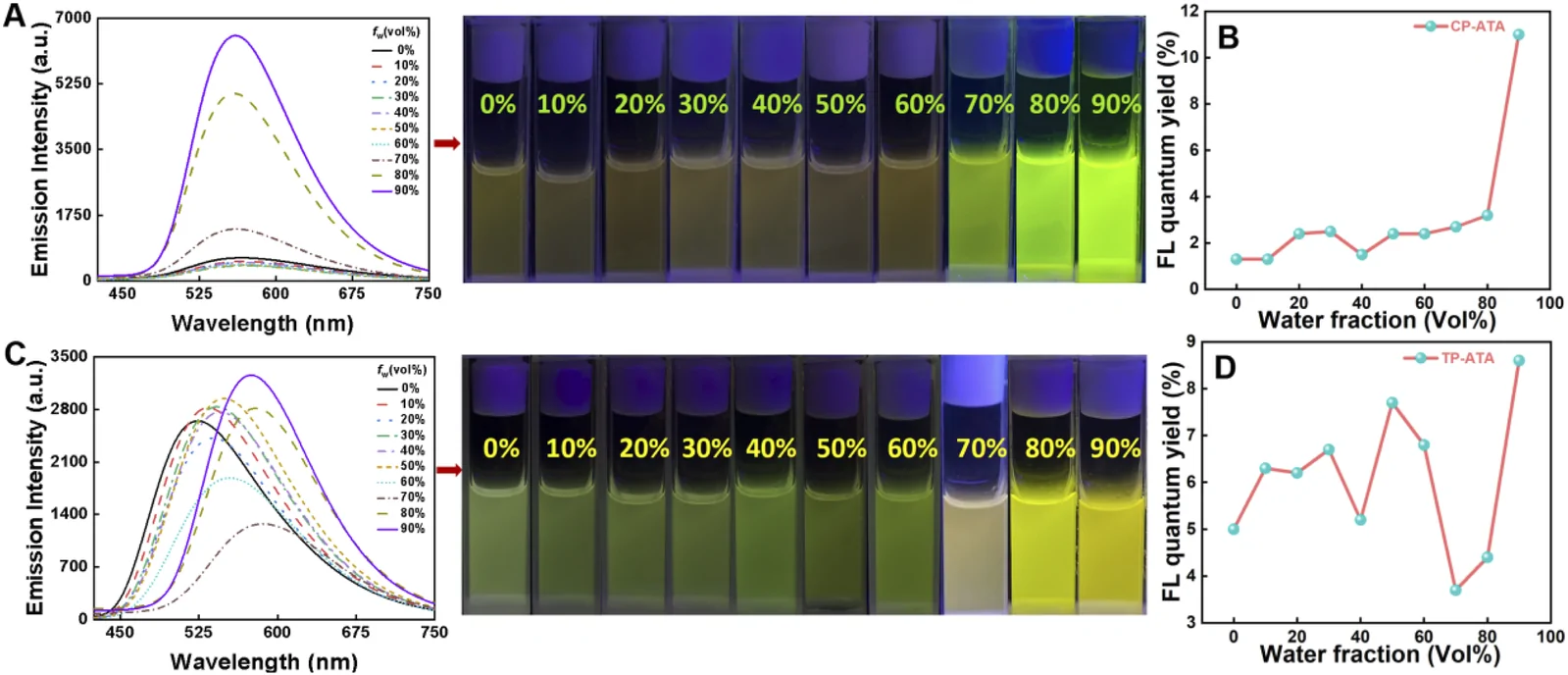
The emission spectrain in THF/H_2_O mixtures with varying water fractions of CP-ATA (A), TP-ATA (B) (*C* = 2.0 × 10^−5^ mol L^−1^) and corresponding photographs under 365 nm UV irradiation; line chart of relative fluorescence quantum yields for CP-ATA (C) and TP-ATA (D).

To further quantitatively verify the AIEE performance of TP-ATA and CP-ATA, absolute fluorescence quantum yield measurements were carried out for these two compounds. The statistical results reveal that the *Φ*_f_ values of CP-ATA and TP-ATA increased remarkably from 1.3% and 5.0% in THF solution to 11.0% and 8.6% in the aggregated state (*f*_w_ = 90%), respectively (Fig. 4B and D). Such prominent luminescence enhancement stems from the formation of nanoparticle aggregates, and their fluorescence lifetimes are measured to be 0.82 ns and 0.78 ns, respectively (Table 1). Compared to previously reported analogous compounds, TP-ATA and CP-ATA exhibit obviously enhanced fluorescence intensity and quantum yield in the aggregated state, along with a remarkable redshift in emission wavelength.^46^ Subsequently, dynamic light scattering (DLS) and scanning electron microscopy (SEM) measurements were further performed to characterize the particle size and microscopic morphology of their nanoaggregates. As illustrated in Fig. 5A and B, the average hydrodynamic diameters of CP-ATA and TP-ATA nanoaggregates are 128 nm and 127 nm, respectively, indicating negligible differences in aggregation size between the two compounds. SEM morphological characterization further verifies their aggregation behaviors (Fig. 5C and D). CP-ATA and TP-ATA self-assembled into spherical aggregates in THF/H_2_O mixture (*f*_w_ = 90%). These consistent characterization results confirm that both luminogens can form stable and densely packed nanoaggregate structures in high-water-fraction mixed solvents, which fundamentally accounts for their prominent AIEE properties.

**Fig. 5:**
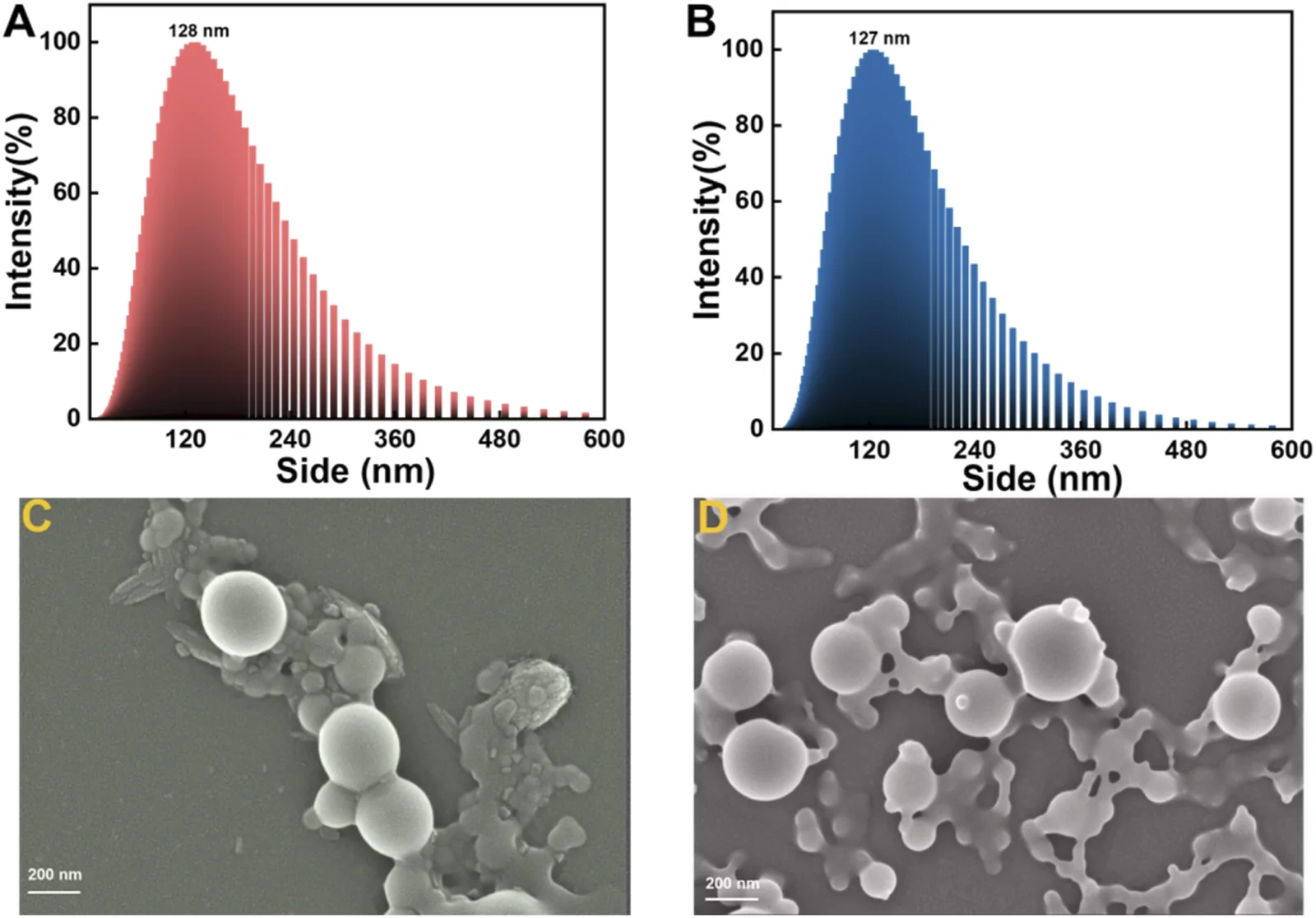
Size distribution and SEM images of CP-ATA (A, C) and TP-ATA (B, D) in THF/H_2_O mixtures (*f*_w_ = 90%).

### 3.4. Mechanofluorochromic property

D–π–A-structured organic molecules have been widely proven to be promising candidates for the construction of high-performance MFC materials. The twisted molecular conformations and unique solid-state luminescence characteristics of such systems can effectively reduce excessive intermolecular contact and facilitate loose molecular packing, which are essential prerequisites for achieving reversible MFC behavior.^47^ Therefore, the MFC performances of CP-ATA and TP-ATA under external mechanical stimulation were systematically investigated, and the corresponding spectral variations are displayed in Fig. 6. The pristine CP-ATA and TP-ATA powders emit bright green and yellowish-green fluorescence under 365 nm UV irradiation, respectively. After mechanical grinding with a mortar, both samples exhibit bright yellow fluorescence. Specifically, CP-ATA presents a 22 nm emission redshift, while TP-ATA shows a more significant redshift of 35 nm (Fig. 6A and B), demonstrating the distinct MFC activity of both compounds. Notably, TP-ATA exhibits superior mechno-responsive performance compared with CP-ATA. This difference originates from the structural characteristics of the donor units. The nitrogen-centered propeller-shaped triphenylamine skeleton provides substantial steric hindrance relative to carbazole in the solid states, endowing TP-ATA with a larger degree of conformational distortion. Such structural features induce loose molecular packing and moderate intermolecular interactions, which are conducive to prominent mechano-responsive spectral shifts.^48^ To evaluate the reversibility of the MFC behavior, dichloromethane (DCM) fuming treatment was performed on the ground samples. The fluorescence color and emission wavelength of both compounds almost fully recovered to their initial states after DCM fuming, verifying the excellent reversibility of their MFC processes. Powder X-ray diffraction (PXRD) measurements were further conducted to clarify the inherent MFC mechanism by characterizing the crystalline structural evolution of the two compounds in pristine, ground, and solvent-fumed states. As shown in Fig. 6C and D, the pristine powders exhibited numerous intense and sharp diffraction peaks, indicating highly ordered crystalline packing. After grinding, the diffraction peaks were significantly weakened or partially disappeared, demonstrating that external mechanical force destroys the ordered crystal structure and transforms the crystalline phase into a disordered amorphous state. Remarkably, after DCM fuming, the attenuated diffraction peaks reappeared and recover their original intensity, confirming the restoration of the crystalline structure. These results verify that the reversible MFC behavior of CP-ATA and TP-ATA originates from the reversible phase transition between the ordered crystalline state and the disordered amorphous state upon mechanical stimulation and solvent annealing.^49^

**Fig. 6:**
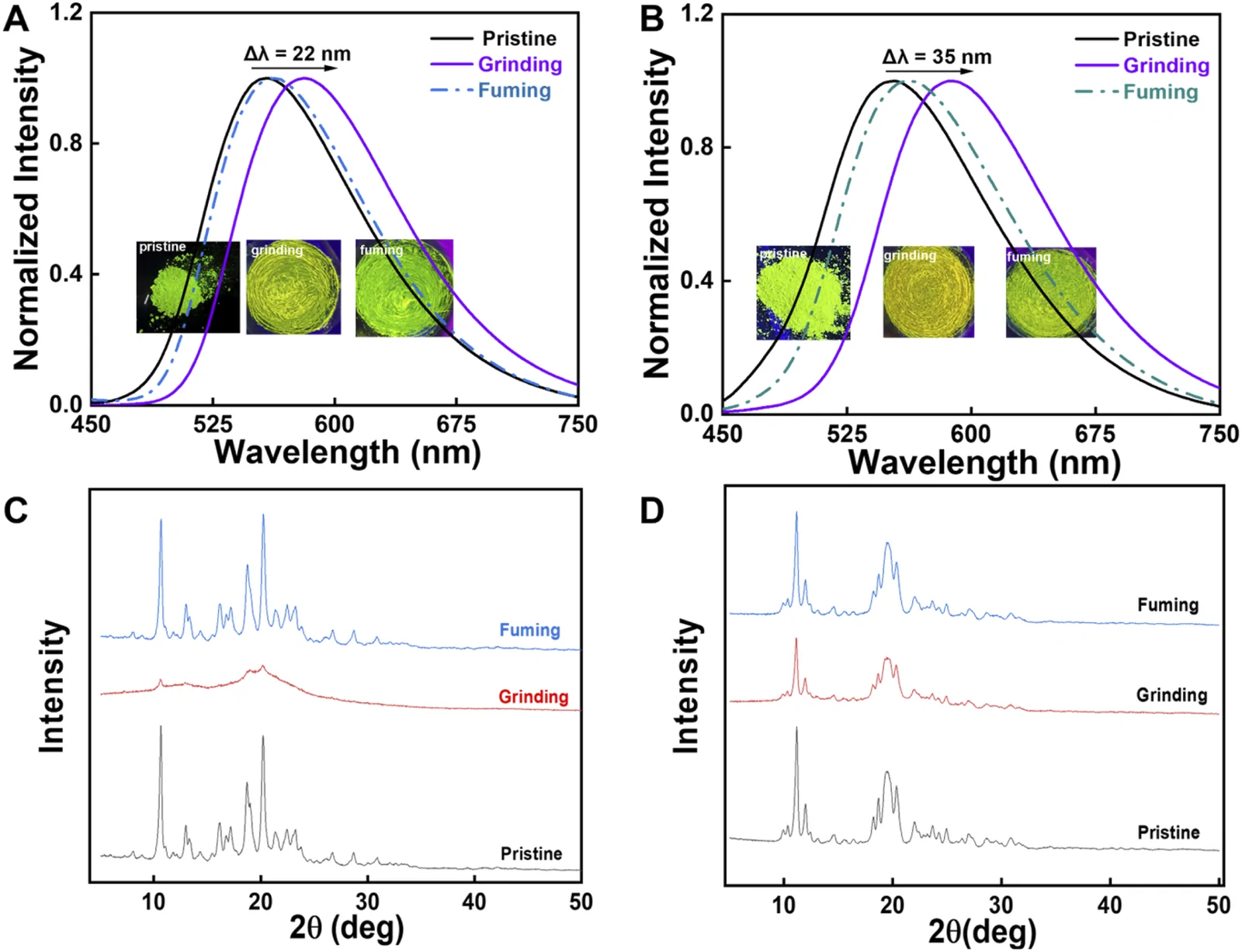
The solid-state PL spectra, XRD curves, and photographic images under 365 nm UV irradiation of CP-ATA and TP-ATA before and after grinding, and after treatment with DCM vapor: CP-ATA (A, C) and TP-ATA (B, D).

### 3.5. Applications

#### 3.5.1. Fluorescent development of latent fingerprints

Fingerprints act as inherent biological identification features and serve as crucial evidence for criminal investigation and forensic analysis. In practical forensic scenarios, fingerprints are formed by skin secretions transferred from fingertips to substrate surfaces. Most of these fingerprint traces are invisible and structurally incomplete, defined as latent fingerprints (LFPs).^50^ Therefore, the development of high-performance luminescent materials for high-precision LFP visualization is urgently needed for forensic applications. Benefiting from unique photophysical properties, AIE/AIEE active fluorophores have emerged as promising candidates for high-contrast LFP imaging. In this work, a facile powder dusting method was adopted for LFP visualization. CP-ATA and TP-ATA were separately mixed with silica at a mass ratio of 1 : 10, and the mixtures were uniformly dispersed in DCM to form homogeneous suspensions. After complete solvent evaporation, two types of luminescent fingerprint developing powders were successfully prepared. Three typical substrates, including glass slides, metal plates, and plastic films, were selected to evaluate the universality and practicability of the as-prepared powders. Taking glass substrates as an example, after LFPs were transferred onto a clean glass slide, the luminescent powder was uniformly sprayed on the fingerprint area, and the excess powder was gently removed using a rubber blower.^51^ Under 365 nm UV irradiation, high-resolution fluorescent LFP images were clearly observed by the naked eye, including level 1 core patterns, level 2 fine ridge details (ridge endings and bifurcations), and even subtle level 3 crease textures (Fig. 7). Subsequently, ImageJ software was utilized to quantify the signal-to-noise ratio (SNR) of the tertiary fingerprint features. The results show that the SNR values obtained by CP-ATA and TP-ATA range from 20–30 dB (Fig. S8, SI), indicating that both of them have good anti-interference performance with negligible background interference. Additionally, clear and distinguishable LFP fluorescent images of CP-ATA and TP-ATA were successfully acquired on metal plates. In contrast, the LFP visualization effect on plastic films was relatively poor. The inherent flexibility and adhesiveness of plastic films easily cause ridge deformation and structural damage during fingerprint transfer and testing, resulting in incomplete and blurred LFP patterns. These results indicate that the developed AIEE luminogens possess excellent practicability and great application potential for high-precision latent fingerprint identification.

**Fig. 7:**
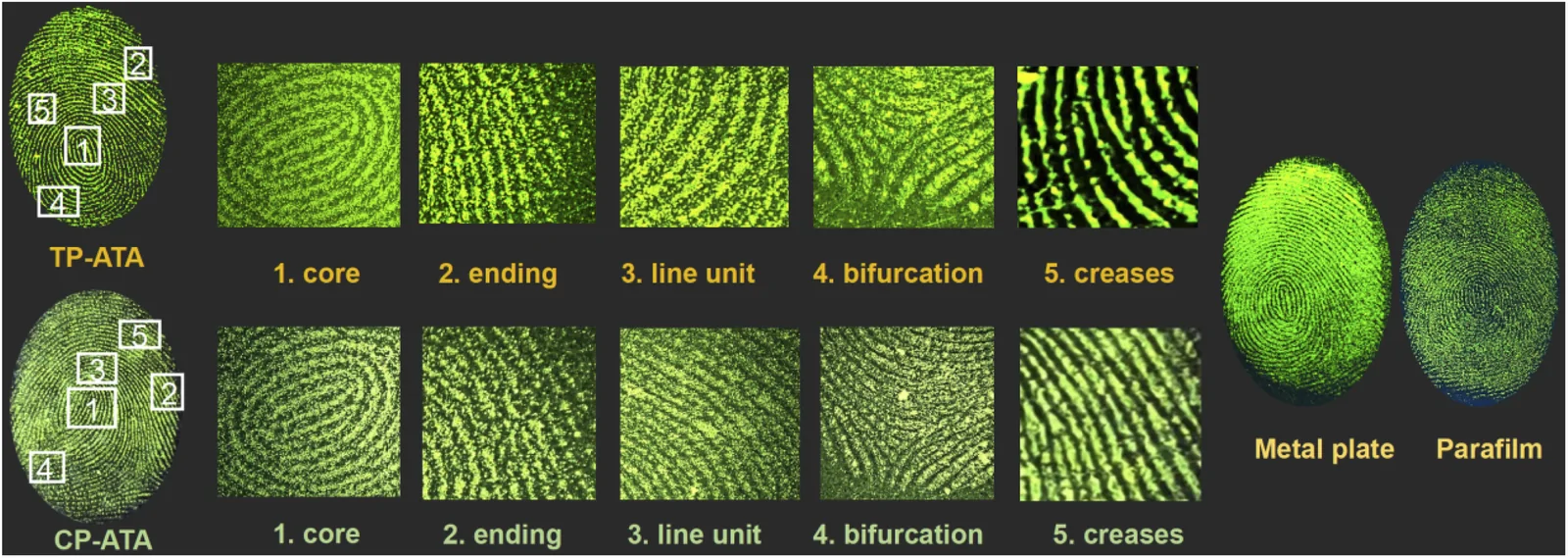
Magnified images of latent fingerprints (LFPs) prepared from two types of powders, showing three-level characteristic details, and corresponding photographs of the developed LFPs on steel plate and plastic film substrates under 365 nm UV irradiation.

#### 3.5.2. Cell cytotoxicity and cell imaging

Given the distinct AIEE characteristics and favorable biological application potential of TP-ATA and CP-ATA, their biocompatibility and cellular imaging performance were systematically evaluated in this study. The cytotoxicity of the two compounds toward 4T1 cells was investigated *via* MTT assay. The cells were incubated with TP-ATA and CP-ATA at gradient concentrations ranging from 5 µM to 30 µM for 24 h. Statistical results revealed that the cell viability remained above 63% even at the maximum treatment concentration (Fig. S9, SI), while a cell viability of over 80% was maintained at 10 µM. Such low cytotoxicity enables further biological imaging investigations. Therefore, confocal laser scanning microscopy (CLSM) imaging was utilized to explore the cellular uptake behavior and fluorescence imaging capacity of the two luminogenic probes. 4T1 cells were independently incubated with 10 µM TP-ATA and CP-ATA. After incubation, the original culture medium was removed, and the cell samples were rinsed with PBS buffer to eliminate unbound probes before replenishing fresh medium. CLSM imaging was performed with an excitation wavelength of 405 nm and an emission collection range of 500–600 nm. As depicted in Fig. 8B1–3, intense yellow fluorescence signals were clearly observed inside CP-ATA-treated 4T1 cells, indicating CP-ATA can effectively penetrate the cell membranes and achieve favorable intracellular accumulation (Fig. 8C1–3). In comparison, TP-ATA tended to aggregate and adhere to the cell membrane surface under identical incubation conditions, and no uniform intracellular fluorescence distribution was observed. These comparative results verify that CP-ATA possesses superior cell permeability and desirable bioimaging performance, endowing it with great potential as a high-performance fluorescent probe for cellular labeling and biological imaging applications.

**Fig. 8:**
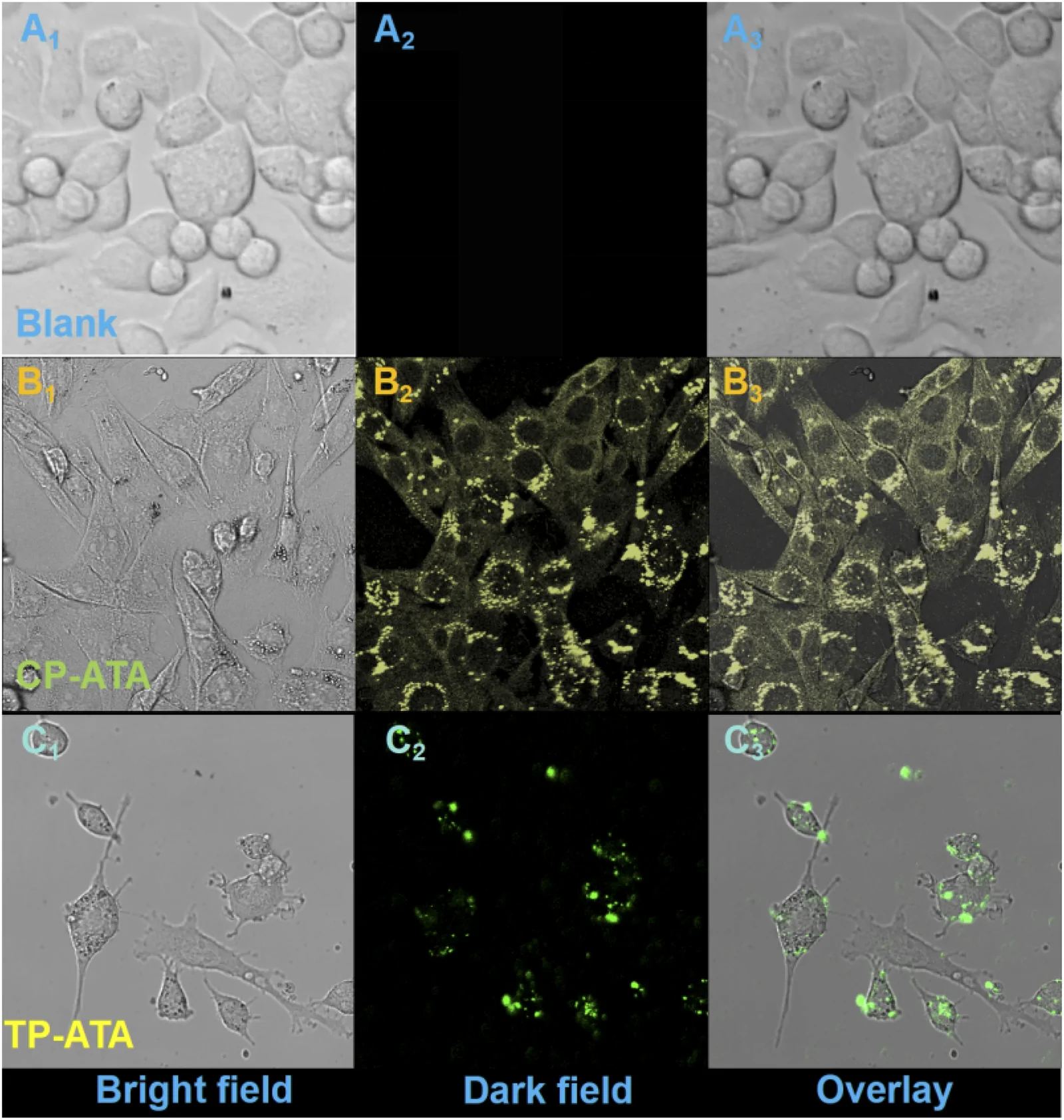
Fluorescent confocal microscopy images of 4T1 cells stained with 10 µM CP-ATA and TP-ATA: (A1–C1) bright-field images; (A2–C2) dark-field images; (A3–C3) overlay of dark and bright field images.

## 4. Conclusions

In summary, two novel AIEE-active luminogens with D–π–A configuration (CP-ATA and TP-ATA) were rationally designed and synthesized. Through the regulation of the electron-donating moieties, controllable tuning of their photophysical performances was achieved. Both luminogens exhibit prominent solvent-dependent solvatochromism originating from intramolecular charge transfer. Combined spectral characterizations in THF/H_2_O mixed solvents and SEM analysis confirmed their typical AIEE features. Furthermore, the two compounds possess reversible mechanofluorochromic behavior enabled by reversible crystalline-amorphous phase transitions, as validated by XRD measurements. Leveraging their intense solid-state luminescence, both luminogens realize high-resolution visualization of latent fingerprints on various substrates. In addition, TP-ATA shows superior cell permeability and desirable bioimaging performance, which enables efficient intracellular imaging in 4T1 cells and demonstrates application potential in the biological field. This work indicates that modulating donor strength and molecular conformation is an effective strategy to optimize the performance and functionality of anthracene-based luminogens. The results provide a valuable reference for the design and development of high-performance multifunctional AIEE materials.

## Conflicts of interest

The authors declare no competing financial interest.

## Supplementary Material

RA-016-D6RA07630K-s001

## Data Availability

The authors confirm that the data supporting the findings of this study are available within the article [and/or] its supplementary information (SI). Supplementary information is available. See DOI: https://doi.org/10.1039/d6ra07630k.
