# Supplementary material for: Synthesis, properties, and applications of novel 9,10-diarylanthracene-based D–A type AIEE fluorescent materials
Source: RSC Adv. 2026 Sep 29;16(45):58483–93. doi: 10.1039/d6ra07630k (PMC13621584; doi:10.1039/d6ra07630k)
Supplement: Supplementary file 1 [file RA-016-D6RA07630K-s001.pdf]

Supplementary Information (SI) for RSC Advances.
This journal is © The Royal Society of Chemistry 2026

†Electronic supporting information

For

Synthesis, properties, and applications of novel 9, 10-diarylanthracene-based D-A type AIEE fluorescent materials

Rui Tian[a], Xing Zhou[a], Shunyang Liu[a], Renjie Wang[a]*, Shouzhi Pu[a,b]*

[a] *Jiangxi Key Laboratory of Organic Chemistry, Jiangxi Science and Technology Normal University, Nanchang, 330013, PR China*

[b] *Jiangxi Key Laboratory of Advanced Ceramic Materials, School of Materials Science and Engineering, Jingdezhen Ceramic University, Jingdezhen 333403, China*

***Corresponding author 1:** E-mail address: bio-wrj@163.com

***Corresponding author 2:** E-mail address: pushouzhi@tsinghua.org.cn

Figure of Contents

Figure S1. NMR spectra data for **Br-ATA** in $CDCl_3$: (A) ^1H-NMR spectrum; (B) ^{13}C-NMR spectrum.

Figure S2. NMR spectra data for **CP-ATA** in $CDCl_3$: (A) ^1H-NMR spectrum; (B) ^{13}C-NMR spectrum.

Figure S3. NMR spectra data for **TP-ATA** in $CDCl_3$: (A) ^1H-NMR spectrum; (B) ^{13}C-NMR spectrum.

Figure S4. HRMS-ESI spectra for **CP-ATA** in acetonitrile.

Figure S5. HRMS-ESI spectra of **TP-ATA** in acetonitrile.

Figure S6. UV-vis absorption spectra of **CP-ATA** (A), **TP-ATA** (B) in different solvents ($C = 2.0 \times 10^{-5}$ mol L^{-1}).

Figure S7. UV-vis absorption spectra of **CP-ATA** and **TP-ATA** in THF/H_2O mixtures with varying volume fractions: (A) **CP-ATA**, (B) **TP-ATA**.

Figure S8. Signal-to-noise ratio distribution maps of the three-level fingerprint features of **CP-ATA** and **TP-ATA**.

Figure S9. Cell viability of 4T1 cells treated with different concentrations (10→30 μM) of **CP-ATA** (A) and **TP-ATA** (B) for 24 h determined by MTT assay.

Table of Contents

Table S1. Photophysical properties of **CP-ATA** in different solvents ($C = 2.0 \times 10^{-5}$ mol L^{-1}).

Table S2. Photophysical properties of **TP-ATA** in different solvents ($C = 2.0 \times 10^{-5}$ mol L^{-1}).

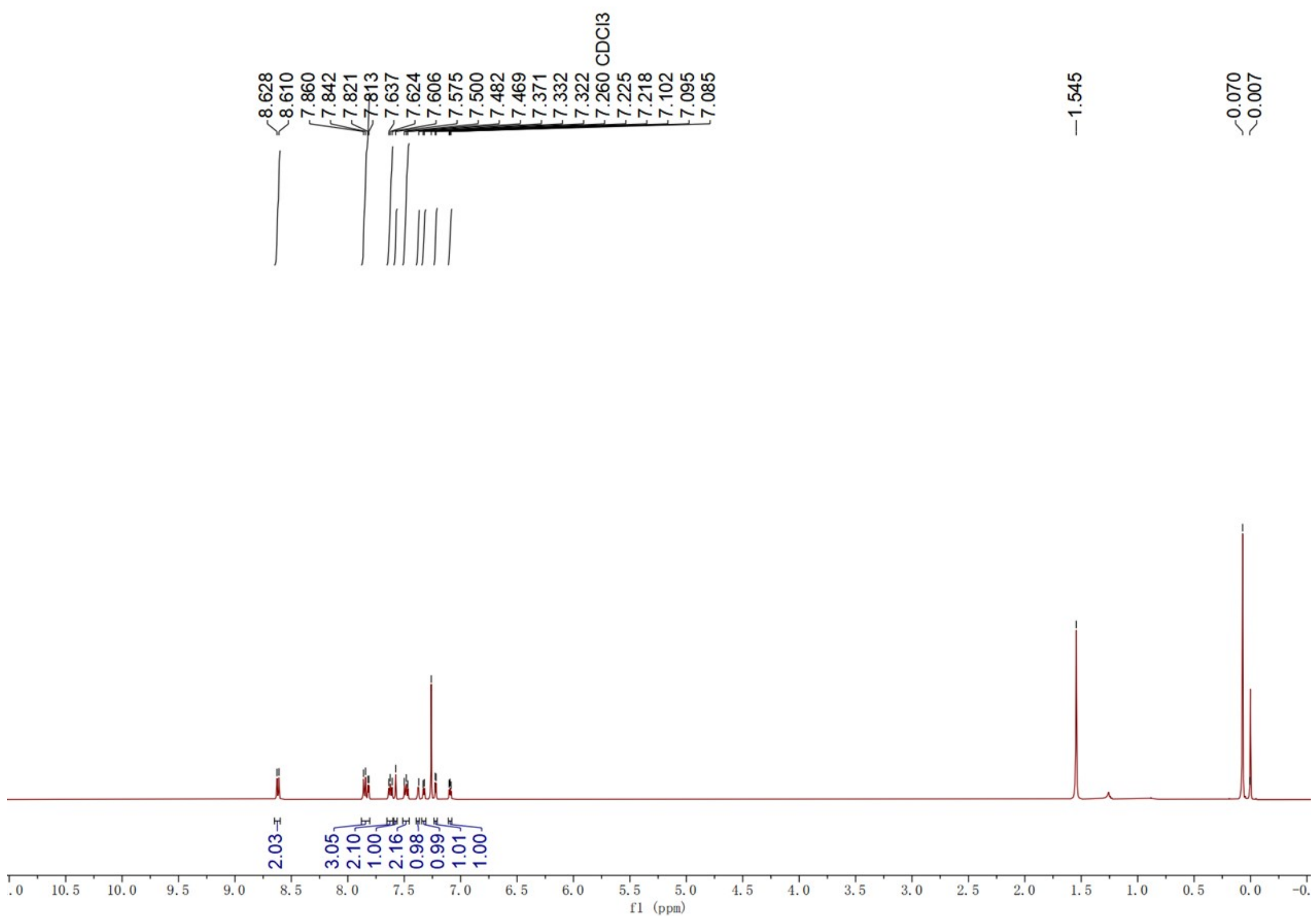

(A)

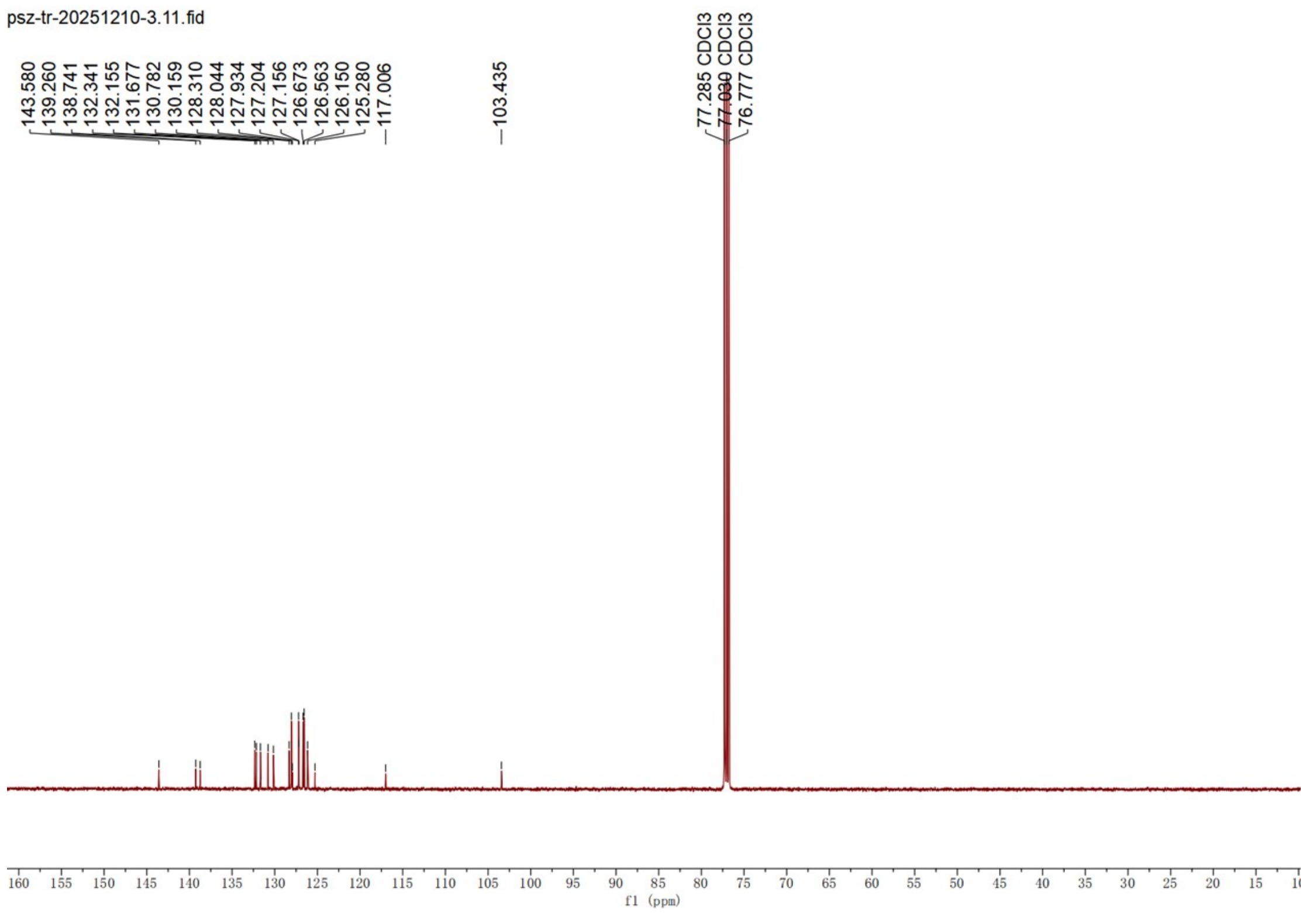

(B)

Figure S1. NMR spectra data for **Br-ATA** in $CDCl_3$: (A) ^{1}H-NMR spectrum; (B) ^{13}C-NMR spectrum.

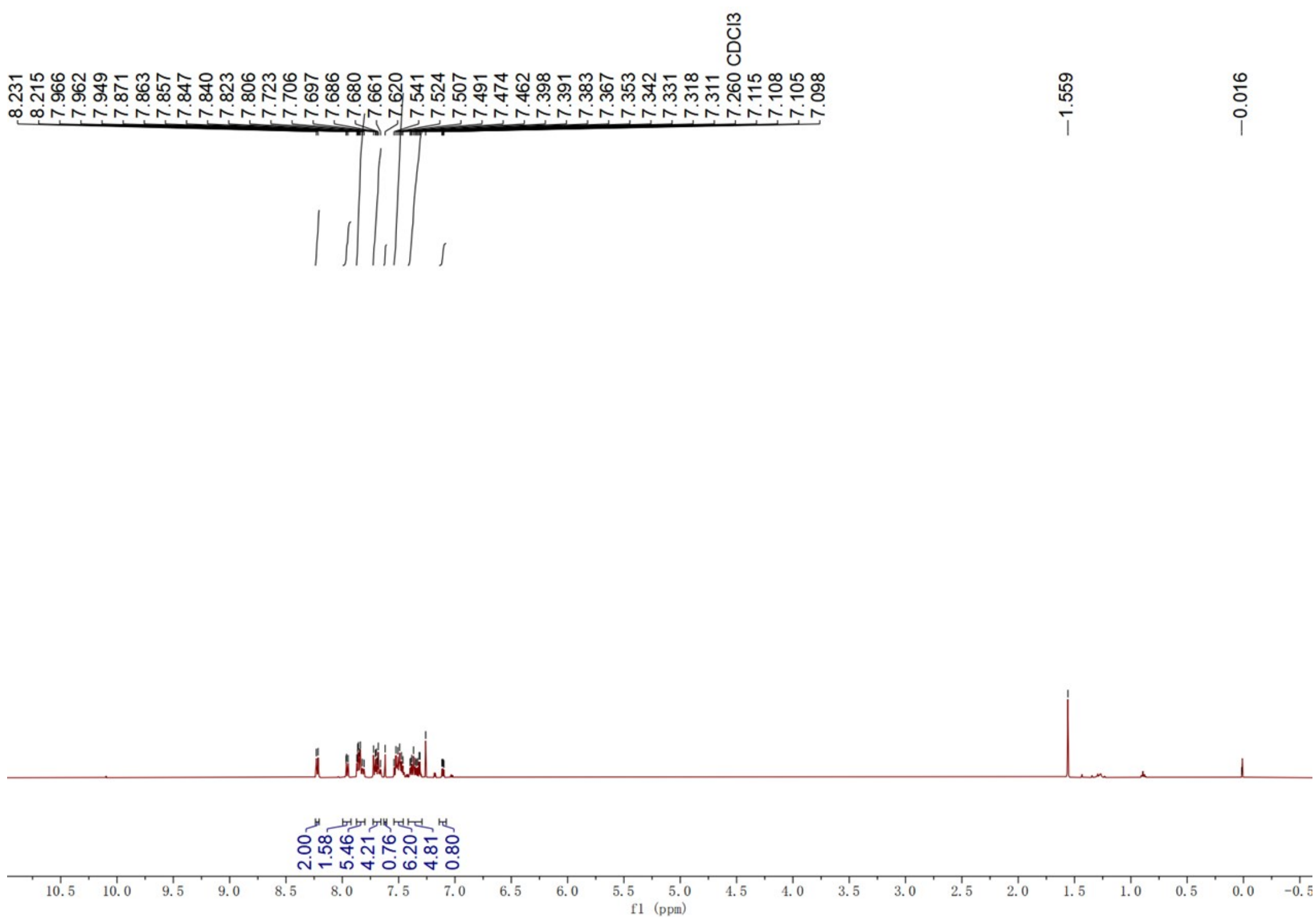

(A)

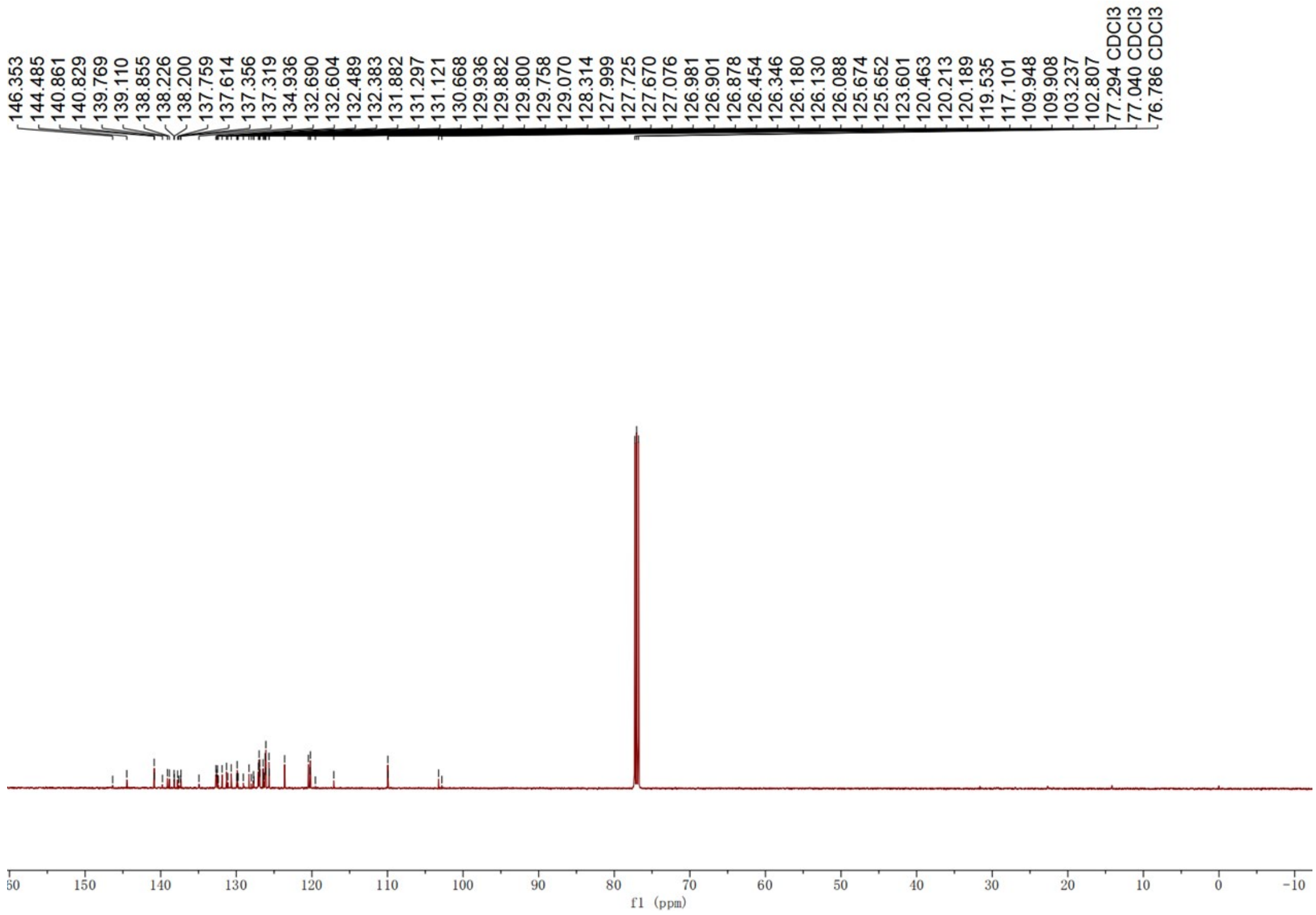

(B)

Figure S2. NMR spectra data for **CP-ATA** in $CDCl_3$: (A) ^{1}H-NMR spectrum; (B) ^{13}C-NMR spectrum.

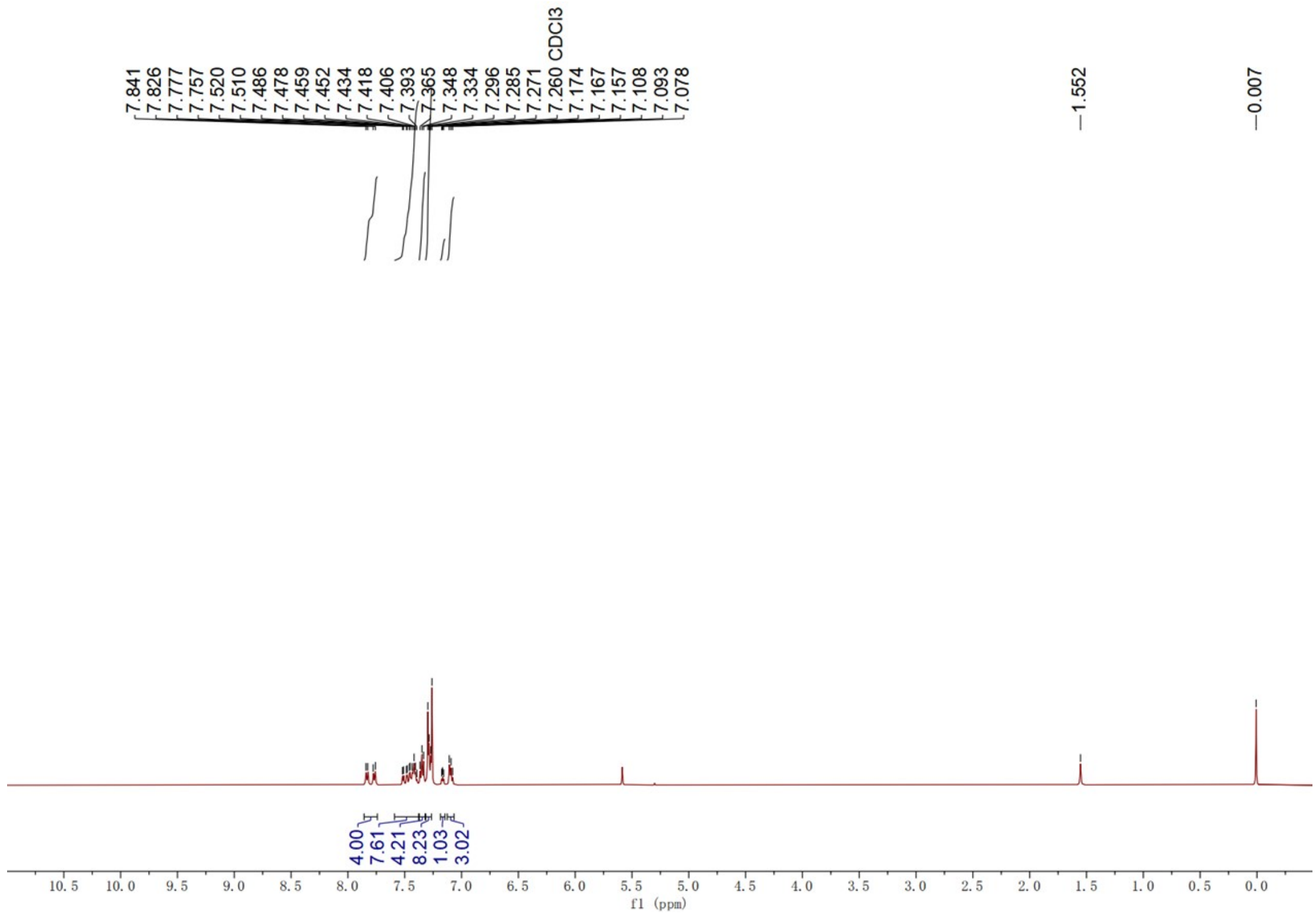

(A)

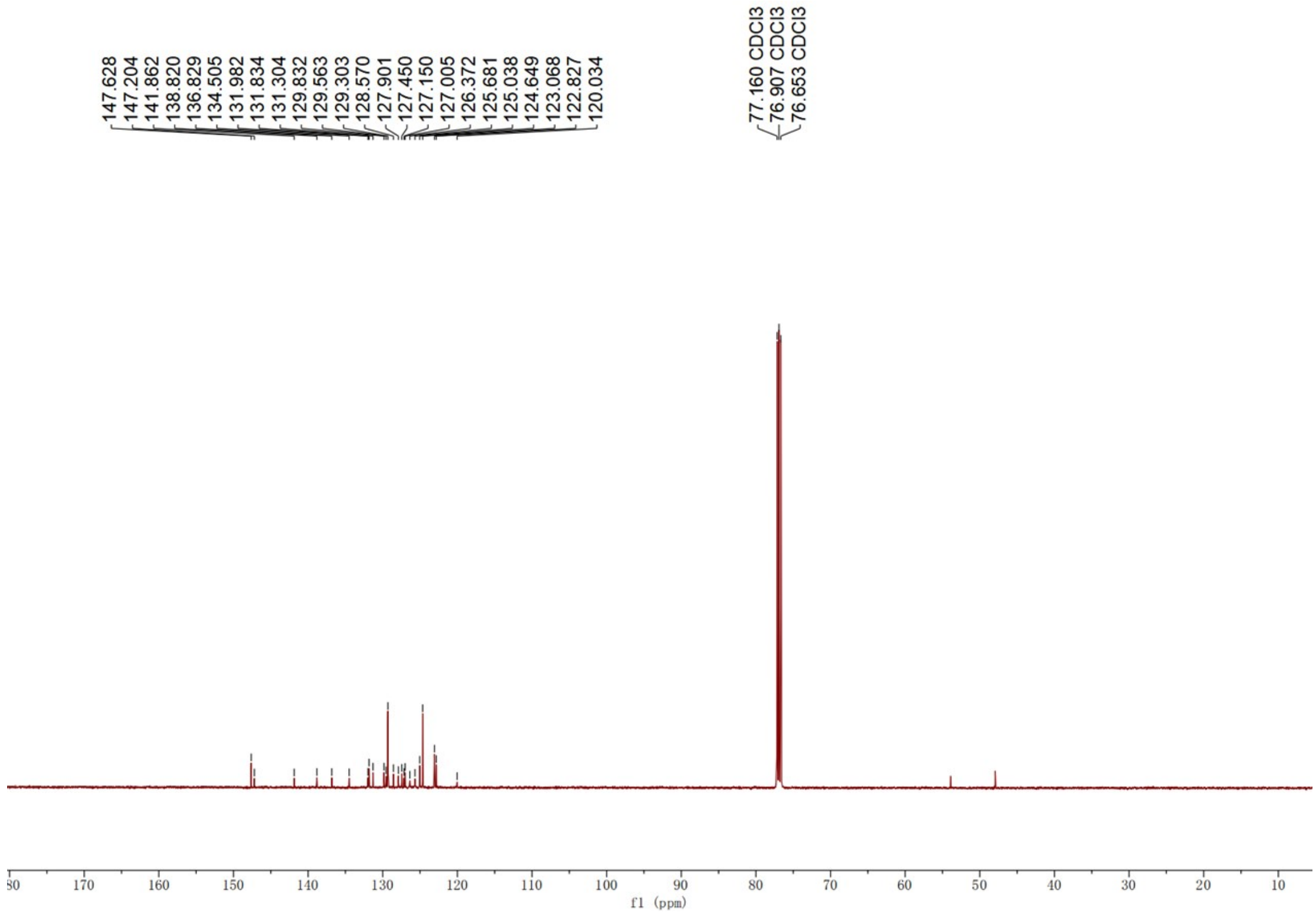

(B)

Figure S3. NMR spectra data for **TP-ATA** in $CDCl_3$: (A) ^{1}H-NMR spectrum; (B) ^{13}C-NMR spectrum.

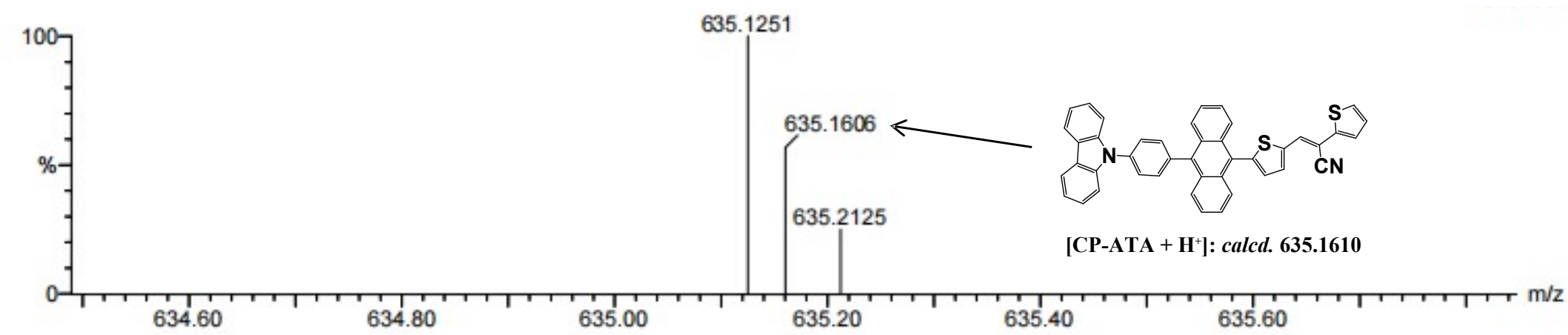

Figure S4. HRMS-ESI spectra for **CP-ATA** in acetonitrile.

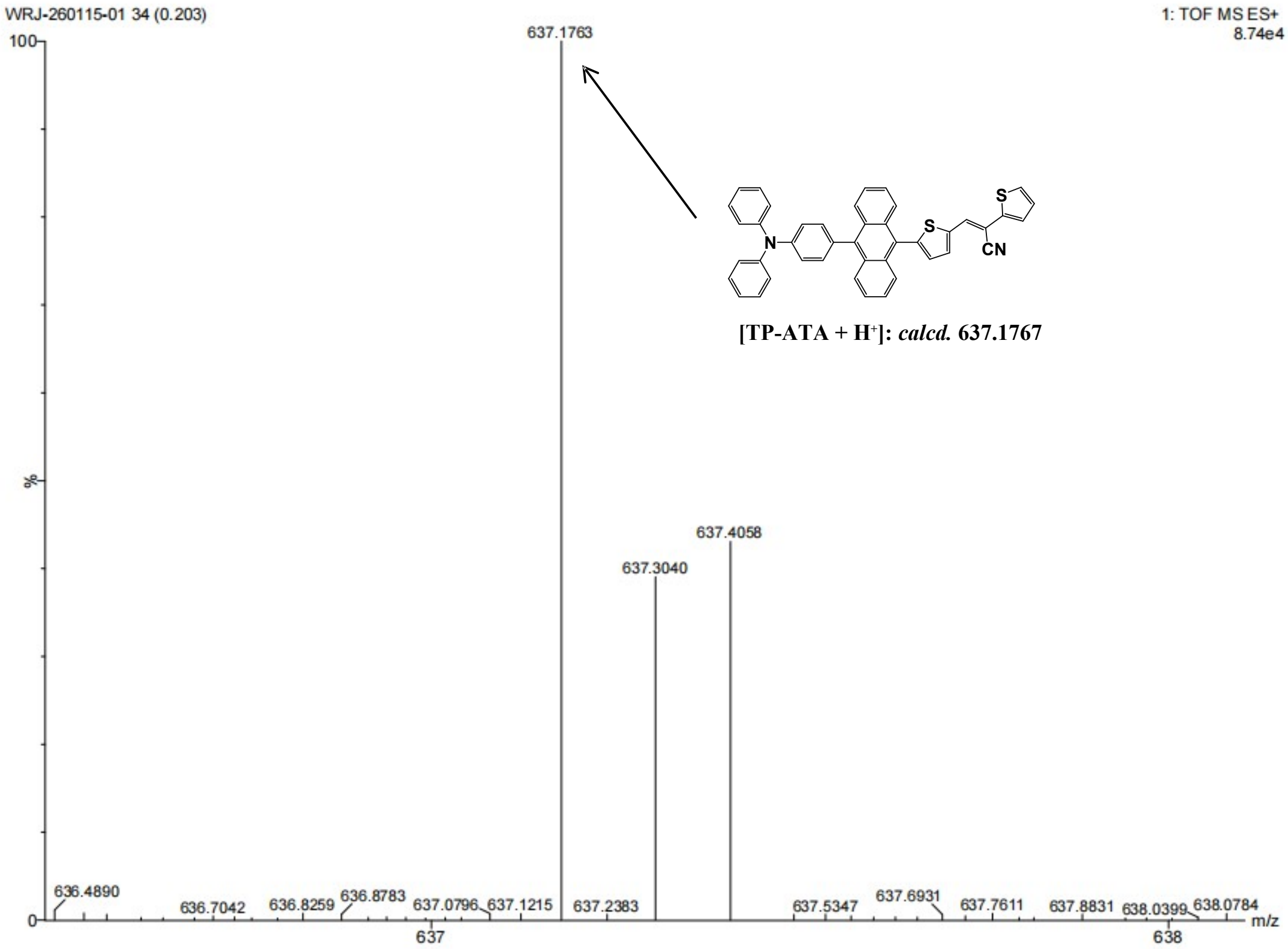

Figure S5. HRMS-ESI spectra for **TP-ATA** in acetonitrile.

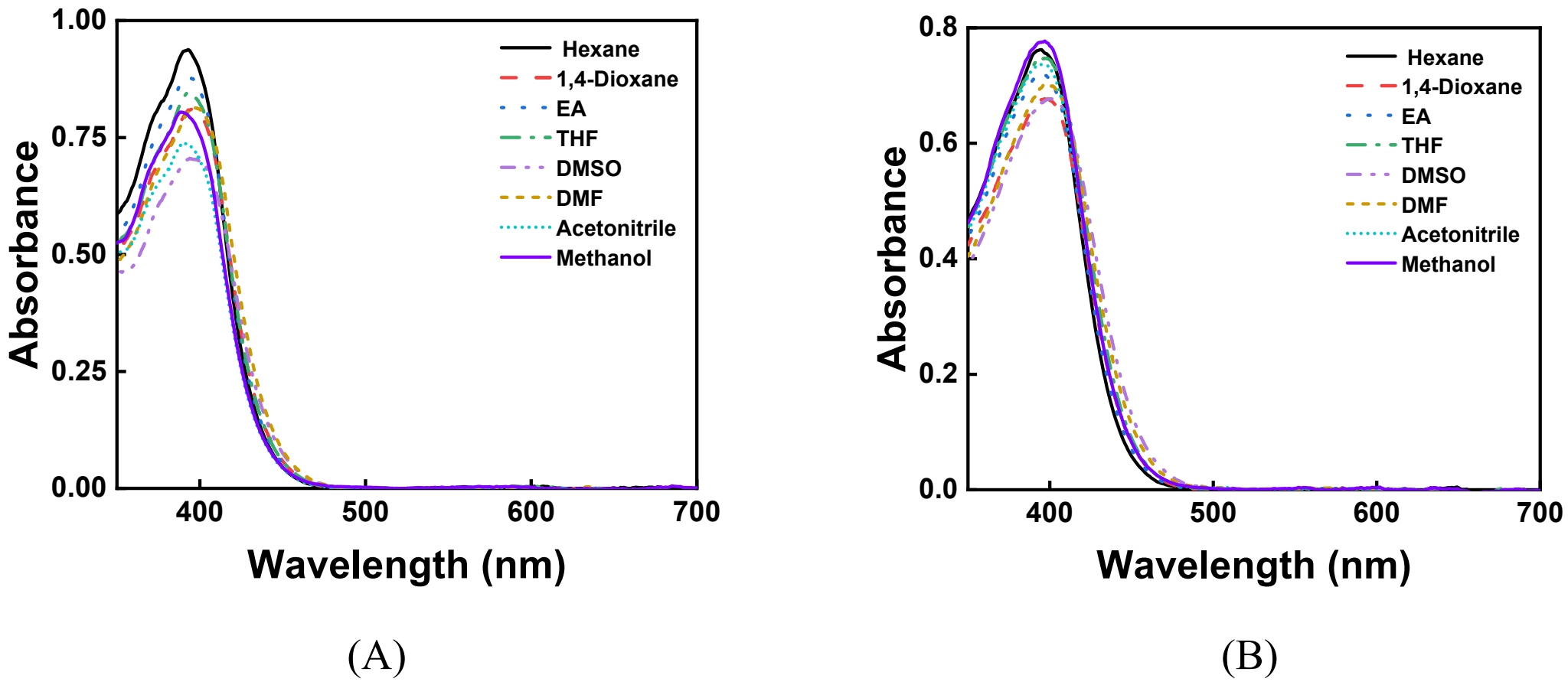

Figure S6. UV-vis absorption spectra of **CP-ATA** (A), **TP-ATA** (B) in different solvents ($C = 2.0 \times 10^{-5}$ mol L^{-1}).

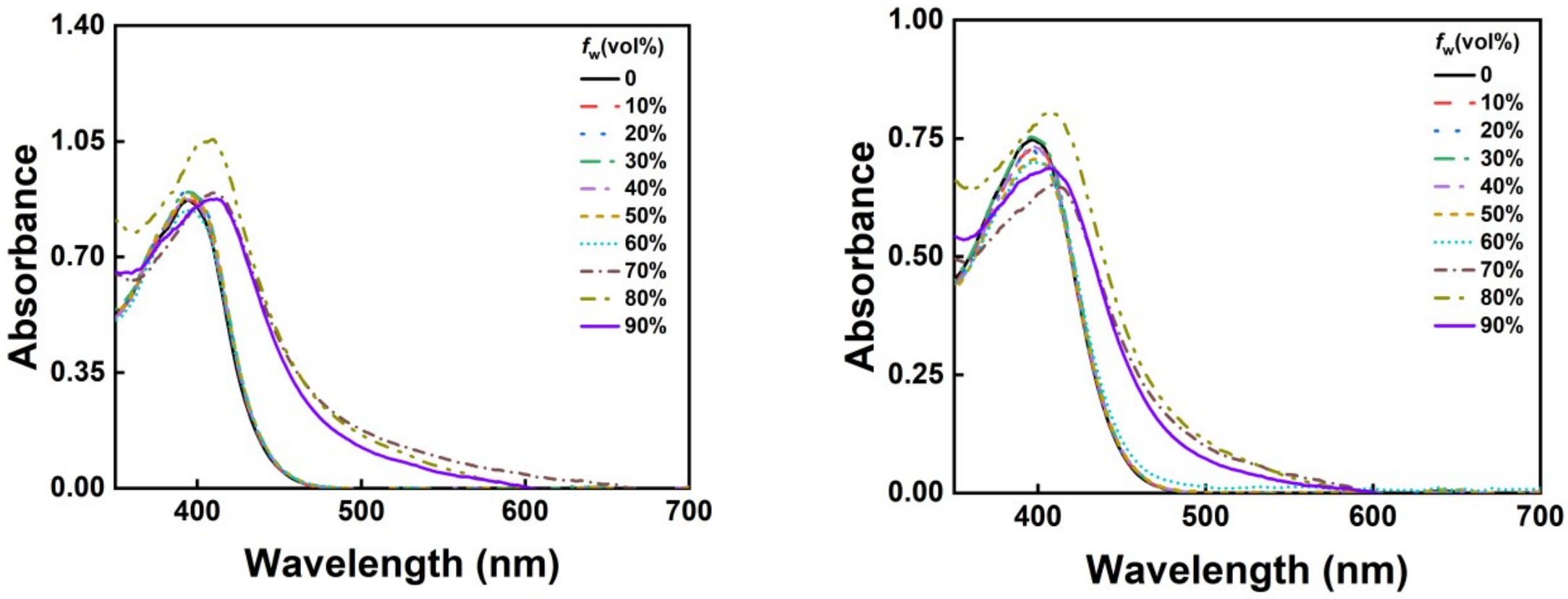

(A) (B)

Figure S7. UV-vis absorption spectra of **CP-ATA** and **TP-ATA** in THF/H_2O mixtures with varying volume fractions: (A) **CP-ATA**, (B) **TP-ATA**.

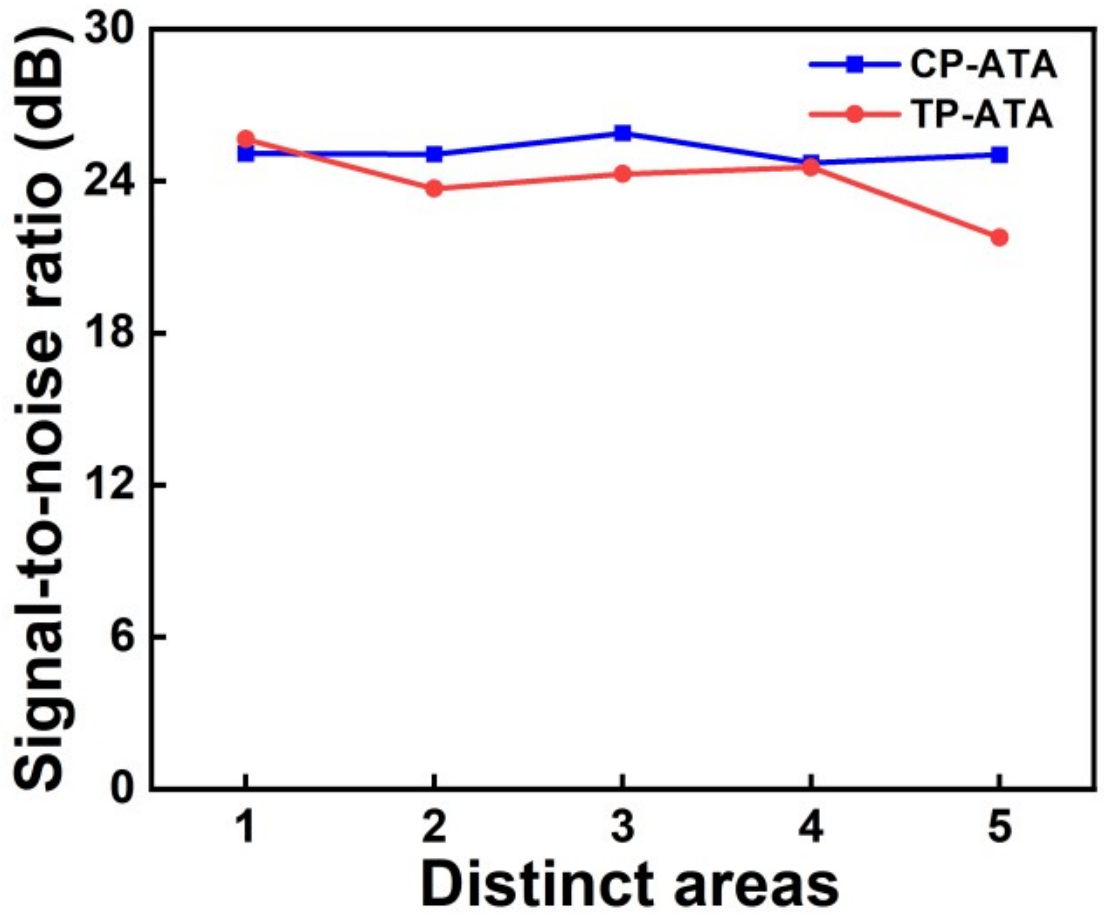

Figure S8. Signal-to-noise ratio distribution maps of the three-level fingerprint features of **CP-ATA** and **TP-ATA**.

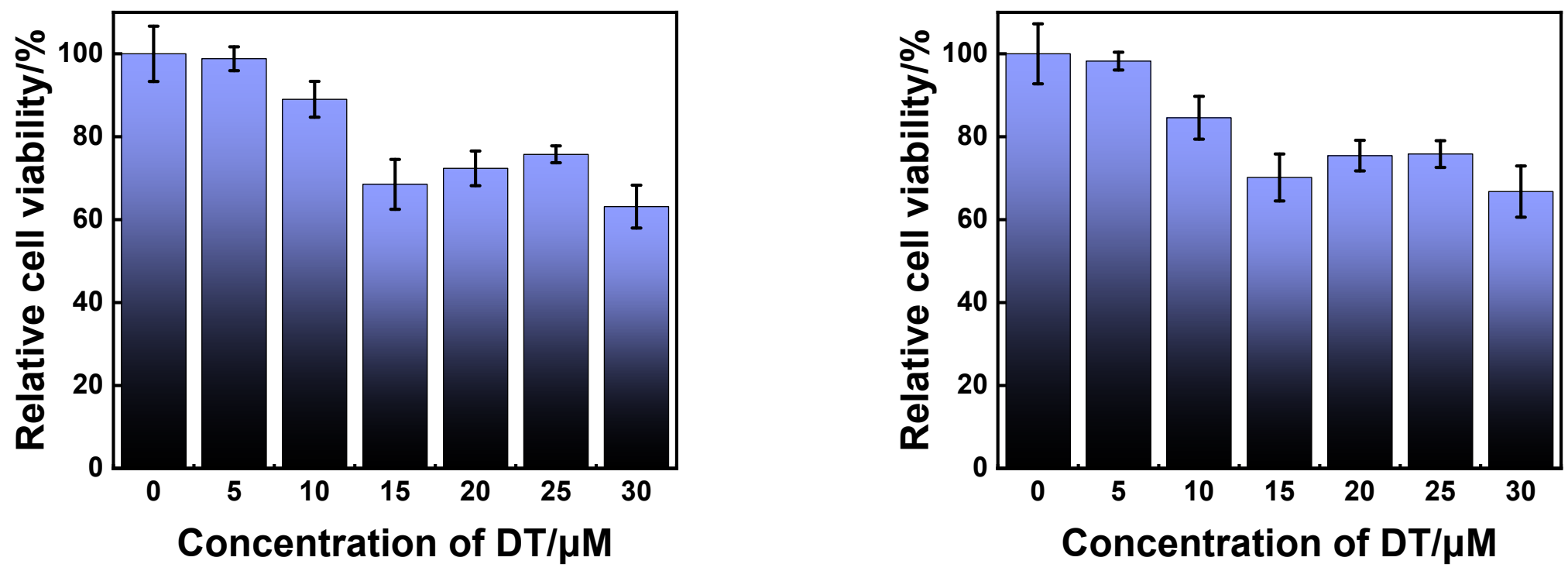

(A) (B)

Figure S9. Cell viability of 4T1 cells treated with different concentrations (10→30 μM) of **CP-ATA** (A) and **TP-ATA** (B) for 24 h determined by MTT assay.

Table S1. Photophysical properties of **CP-ATA** in different solvents ($C = 2.0 \times 10^{-5}$ mol L^{-1}).

Compound	Solvent	Δf^a	λ_{max} (nm)^b	λ_{em} (nm)^c	Stokes shift (Δ/cm^{-1})	$\Phi_{f\,rel}{}^d$	ε^e (mol^{-1} L cm^{-1})
**CP-ATA**	Hexane	0.001	393	550	7263	0.010	4.69×10^4
	1,4-Dioxane	0.164	398	556	7140	0.022	4.05×10^4
	EA	0.200	392	564	7779	0.011	4.40×10^4
	THF	0.209	393	567	7808	0.011	4.23×10^4
	DMSO	0.264	394	589	8402	0.019	3.53×10^4
	DMF	0.267	396	585	8158	0.012	4.07×10^4
	CH_3CN	0.305	391	589	8597	0.009	3.69×10^4
	CH_3OH	0.309	389	576	8345	0.007	4.02×10^4

^{a} Directed polarizability; ^{b} absorption wavelength; ^{c} the wavelength of emission maximum; ^{d} Relative Fluorescence Quantum Yield ($\Phi_{f,\,anthracene} = 0.27$); ^{e} molar extinction coefficient.

Table S2. Photophysical properties of **TP-ATA** in different solvents ($C = 2.0 \times 10^{-5}$ mol L^{-1}).

Compound	Solvent	Δf^{a}	λ_{max} (nm)^{b}	λ_{em} (nm)^{c}	Stokes shift (Δ/cm^{-1})	$\Phi_{f\,rel}{}^{d}$	ε^{e} (mol^{-1} L cm^{-1})
**TP-ATA**	Hexane	0.001	395	505	5514	0.025	3.81×10^4
	1,4-Dioxane	0.164	399	515	5645	0.073	3.39×10^4
	EA	0.200	394	522	6223	0.036	3.60×10^4
	THF	0.209	397	523	6068	0.039	3.74×10^4
	DMSO	0.264	403	586	7749	0.043	3.39×10^4
	DMF	0.267	400	568	7394	0.033	3.51×10^4
	CH_3CN	0.305	397	581	7977	0.029	3.69×10^4
	CH_3OH	0.309	397	550	7007	0.018	3.89×10^4

^{a} Directed polarizability; ^{b} absorption wavelength; ^{c} the wavelength of emission maximum; ^{d} Relative Fluorescence Quantum Yield ($\Phi_{f,\ anthracene} = 0.27$); ^{e} molar extinction coefficient.
